# RAB6B is a potential prognostic marker and correlated with the remolding of tumor immune microenvironment in hepatocellular carcinoma

**DOI:** 10.3389/fphar.2022.989655

**Published:** 2022-09-02

**Authors:** Hao Peng, Erwei Zhu, Jitao Wang, Xuanlong Du, Chonggao Wang, Meng Yang, Yewei Zhang

**Affiliations:** ^1^ Medical School, Southeast University, Nanjing, China; ^2^ The Second People’s Hospital of Lianyungang (The Oncology Hospital of Lianyungang), Lianyungang, China; ^3^ Xingtai Institute of Cancer Control, Xingtai People’s Hospital, Xingtai, China; ^4^ State Key Laboratory of Complex Severe and Rare Diseases, Department of Ultrasound, Peking Union Medical College Hospital, Chinese Academy of Medical Sciences and Peking Union Medical College, Beijing, China; ^5^ Hepatopancreatobiliary Center, The Second Affiliated Hospital of Nanjing Medical University, Nanjing, China

**Keywords:** HCC, immune infiltration, RAB6B, CAFs, TME

## Abstract

**Backgrounds:** Hepatocellular carcinoma (HCC) is the most common type of primary liver cancer and the second leading cause of death among all cancers. The Ras-associated binding (Rab) proteins constitute the largest family of the Ras superfamily of small GTPases, which mainly mediate membrane trafficking processes. RAB6B is a member of Rab GTPases, and it has been found to be dysregulated in various tumors. However, the clinical significance, correlations with immune cells, and stroma infiltration of RAB6B in HCC remain unclear.

**Methods:** RAB6B mRNA and protein expression in HCC were examined using the TIMER, HCCDB, UALCAN, and HPA databases. The genetic alterations of RAB6B were analyzed by cBioPortal and COSMIC databases. The correlations between RAB6B and tumor-infiltrating immune cells and cancer-associated fibroblasts were explored by using TIMER, TISIDB, and GEPIA databases. Co-expression networks of RAB6B were investigated based on LinkedOmics. Drug sensitivity was analyzed through the GDSC and CTRP databases. RAB6B was knocked down with siRNA in HCC cell lines. EdU assay was performed to detect the cell proliferation ability, flow cytometry was used to compare the differences in the ability of apoptosis, and MTT was used to evaluate the drug sensitivity *in vitro*.

**Results:** RAB6B mRNA and protein expression were upregulated in the HCC tissues. Kaplan–Meier and Cox regression analyses suggested that highly expressed RAB6B was an independent prognostic factor for poor survival in HCC patients. Moreover, we found that RAB6B expression was positively correlated with the infiltration of immune cells in HCC, including some immunosuppressive cells, chemokines, and receptors, meanwhile RAB6B expression was associated with CD8+T cells exhaustion, resulting in an immunosuppressive microenvironment. Additionally, functional enrichment analysis indicated that RAB6B may be involved in ECM remodeling in the TME, and RAB6B expression was positively associated with CAFs infiltration. Furthermore, RAB6B presented a positive association with sensitivity to GDSC and CTRP drugs. RAB6B knockdown inhibited the cell proliferation and promoted apoptosis and sensitivity to cisplatin of HCC cells *in vitro*.

**Conclusion:** Our study revealed that RAB6B is a potential biomarker for poor prognosis in HCC patients and correlates with the formation of the immunosuppressive microenvironment in HCC.

## Introduction

Hepatocellular carcinoma (HCC), the most common type of liver cancer, mainly develops from chronic liver diseases, such as hepatitis B infection, hepatitis C infection, and liver fibrosis (Craig et al., 2020). Most HCC patients were diagnosed at an advanced stage and lost the opportunity for optimal surgical resection, so it is urgent to discover new reliable HCC markers for diagnosis and prognosis of HCC. Currently, emerging research focus on the role of the tumor microenvironment (TME) on tumor progression. TME or tumor stroma includes various tumor-associated immune cells, vascular endothelial cells, cancer-associated fibroblasts (CAFs), and extracellular matrix (ECM). The stroma components continuously communicate with tumor cells to provide nutrients and growth factors for tumor occurrence and development (Lu et al., 2019). Moreover, it has been recently reported that oncogenic mutations in tumor cells can also influence tumor-stroma interactions by altering the expression of chemokines, cytokines, immune checkpoint molecules, and ECM remodeling in the TME, thereby fostering a more conducive microenvironment for tumor growth (Nishida and Kudo, 2018).

The Rab GTPase is a family of proteins that mediate membrane trafficking, and distinct Rab proteins located in specific organelles are involved in the regulation of cell growth, survival, and death (Gopal Krishnan et al., 2020). Additionally, accumulating studies have reported that dysregulated expression or mutation of several Rab proteins may affect tumor migration, invasion, metastasis, stromal cell communication, and drug resistance in HCC (Yang et al., 2021). RAB6B is an isoform protein of the RAB6 subfamily, mainly located in the Golgi complex (Opdam et al., 2000). Recently, studies have shown that RAB6B expression in gastric cancer can promote tumor cells proliferation, while RAB6B is low expressed and associated with poor prognosis in pancreatic cancer patients, suggesting that the role of RAB6B is context-specific (Anand et al., 2020; Zhao et al., 2020). However, the role and mechanism of RAB6B expression on the prognosis, progression, and immune infiltration in HCC have not been investigated.

In this study, we comprehensively investigated the expression level, genetic alterations, and prognostic and diagnostic significance of RAB6B in HCC patients in various public databases. Moreover, the association between RAB6B and infiltrating immune cells and CAFs in TME was also analyzed. To explore the potential biological functions of RAB6B, the functional enrichment analysis of co-expressed genes with RAB6B was performed. Meanwhile, we analyzed the drug responsiveness of RAB6B in HCC through GDSC and CTRP databases. Finally, a series of functional assays were performed to further evaluate the roles of RAB6B knockdown on HCC cell proliferation, apoptosis, and drug sensitivity.

## Materials and methods

### RAB6B gene expression analysis

TIMER2 (http://timer.cistrome.org/) was used to examine the expression of RAB6B in liver cancer and corresponding normal liver tissues through The Cancer Genome Atlas (TCGA) data (Li et al., 2017). The HCCDB database (http://lifeome.net/database/hccdb/home.html), which contains 15 public HCC datasets from Gene Expression Omnibus (GEO), TCGA, and International Cancer Genome Consortium (ICGC) databases, was used to further validate the differential expression of RAB6B in liver cancer and normal tissues (Ferlay et al., 2015). Besides, the mRNA and protein expression levels of RAB6B were also detected from the UALCAN (http://ualcan.path.uab.edu) (Chandrashekar et al., 2017). Immunohistochemistry (IHC) analysis of RAB6B in liver cancer samples and normal liver samples was explored from the Human Protein Atlas (HPA) (http://www.proteinatlas.org/) (Asplund et al., 2012). Correlation analysis of RAB6B and clinical parameters, including T stage and histological grade were performed with R using the ggplot2 package.

### Survival prognosis analysis and nomogram establishment

The Kaplan- Meier plotter database (http://kmplot.com/analysis/) was used to evaluate the association of RAB6B expression with the survival of the HCC cohort (Lanczky and Gyorffy, 2021). Furthermore, the prognostic value of RAB6B in HCC *via* univariate and multivariate Cox regression analysis was analyzed by using the R survival package. To assess the diagnostic value of RAB6B in HCC, a receiver operating characteristic (ROC) curve was performed and the area under the curve (AUC) was calculated by using the “pROC package.” Besides, to predict the survival risk of HCC patients, the prognostic nomograms were constructed based on the multivariate Cox model by using the R package “rms”.

### Genetic alteration analysis

The cBioPortal database (http://cbioportal.org) was used to analyze the RAB6B gene alteration. Liver Hepatocellular Carcinoma (LIHC) (TCGA, Firehose Legacy) dataset was selected for subsequent analysis (Gao et al., 2013). The Catalogue of Somatic Mutations in Cancer (COSMIC) database (https://cancer.sanger.ac.uk/cosmic) was used to explore the different mutant types of RAB6B in HCC (Forbes et al., 2015).

### Immune infiltration analysis

The relationship between RAB6B expression and ImmuneScore, StromalScore, and ESTIMATEScore was analyzed based on R packages “estimate” through the website of “http://sangerbox.com/Tool.” TISIDB (http://cis.Hku.hk/TISIDB/) database was used to analyze the correlation between the RAB6B expression and various tumor-infiltrating immune cells, immunoinhibitors, immunostimulators, chemokines, and receptors (Ru et al., 2019). The correlation between RAB6B expression and infiltrating levels of regulatory T cells (Tregs), myeloid-derived suppressor cells (MDSC), Macrophages, and cancer-associated fibroblasts cells (CAFs) in HCC was analyzed by “gene modules” from TIMER2.0 database. Meanwhile, analysis of RAB6B expression combined with various immune infiltrating cells to predict patient survival was performed by “outcome modules” through the TIMER2.0 database. GEPIA2 (http://gepia2.cancer-pku.cn/) was used to compare the correlation between RAB6B expression and TGFB1, and IL10 (Tang et al., 2019). RAB6B expression was further evaluated the expression differences between different immune cell subsets by using single-cell RNA-sequencing results of six HCC patients through http://cancer-pku. Cn:3838/HCC/(Zhang et al., 2019).

### Functional enrichment analysis

The LinkedOmics database (http://www.linkedomics.org/login. php) was used to search for the differentially expressed genes related to RAB6B in HCC using the LinkFinder module. Genes positively and negatively associated with RAB6B were analyzed and visualized by volcano plot and heat maps (Vasaikar et al., 2018). Gene Ontology (GO) analysis and Kyoto Encyclopedia of Genes and Genomes (KEGG) pathway enrichment analysis were performed to annotate the differentially expressed genes related to RAB6B.

### Correlation between RAB6B and drug response

Gene Set Cancer Analysis (GSCA) (http://bioinfo.life.hust.edu.cn/GSCA/#/) was used to explore the correlation between the RAB6B expression and drug sensitivity. The module “GDSC drug sensitivity and expression correlation” and “CTRP drug sensitivity and expression correlation” were selected to analyze the correlation. The detailed information on the differential drug was annotated from Genomics of Drug Sensitivity in Cancer (GDSC) and Cancer therapeutics Response Portal (CTRP).

### Cell culture and siRNA transfection

Human liver cancer cell lines (Huh7, MHCC97L, Hep3B, and HepG2) were purchased from the Shanghai Institute for Biological Science, Chinese Academy of Science (Shanghai, China), SMMC7721 cells were purchased from Xiamen Immocell Biotechnology Co., Ltd, all cells were cultured in Dulbecco’s modified Eagle’s medium (DMEM, Gibco, Thermo Fisher Scientific, United States) containing 10% fetal bovine serum (FBS, Invitrogen, United States) and 1% penicillin-streptomycin at 37°C in humidified air with 5% CO2. The RAB6B-specific siRNAs and negative control (NC) were designed from RIBOBIO (Guangzhou, China). Transfection was performed with Lipofectamine 2000 (Invitrogen, United States), and cells were collected for the further experiment after transfecting for 48 h.

### qRT-PCR

Total RNA from cultured HCC cell lines was extracted with Trizol reagent (Invitrogen, United States) according to the manufacturer’s instructions. 1 μg RNA samples were reverse transcribed into cDNA using a SweScript RT I First Strand cDNA Synthesis Kit (Servicebio, China). The qRT-PCR was carried out using 2 × SYBR Green qPCR Master Mix (High ROX) (Servicebio, China). All primers were listed as follows: RAB6B Forward: AGA​GGC​AGA​TAA​CCA​TCG​AGG, Reverse: CTT​CGC​ACT​GGT​CTC​AAT​GAA. GAPDH Forward: GGA​GCG​AGA​TCC​CTC​CAA​AAT, and Reverse: GGC​TGT​TGT​CAT​ACT​TCT​CAT​GG. GAPDH was utilized as the internal control.

### Ethynyl deoxyuridine incorporation assay

After transfection with siRAB6B for 48 h, SMMC7721 and MHCC97L cells were seeded in 96-well plates (2,000 cells/well). After incubation overnight, cells were labeled with EdU and performed according to the manufacturer’s instructions (Beyotime, China). The cells were visualized with a Zeiss Axio Observer microscope, and images were captured in at least three random fields for further analysis.

### Cell apoptosis assays

After transfection with siRAB6B for 48 h, SMMC7721 and MHCC97L cells were seeded in 24-well plates (20,000 cells/well). Cells were cultured overnight and induced apoptosis with 20 µM cisplatin for 18 h. Then, cells were isolated with EDTA-free trypsin, washed with cold PBS three times, and resuspended in a binding buffer. After incubation with PI and Annexin V-FITC (Vazyme, China) in dark for 10 min, the cell apoptosis was examined through the flow cytometer (Thermo AttuneNxt, United States) and analyzed by FlowJo software.

### MTT assay

Transfected cells were seeded into the 96-well plate (4,000 cells/well), after overnight attachment, the medium was changed to 100 µl fresh medium with dosage cisplatin (Selleck, China) and cultured for another 24 h or 48 h 5 mg/ml MTT (Beyotime, China) was added to each well and continued to incubate for 4 h, then discarded the supernatant, added 150 µl DMSO, and detected the OD value at 490 nm by a microplate reader (ThermoFisher, United States).

### Statistical analysis

The Wilcoxon test was used to examine the RAB6B mRNA expression levels between pairs of groups. Logistic regression was conducted to analyze the association of the RAB6B expression and clinicopathological parameters. The Kaplan–Meier method and log-rank tests were performed to analyze the overall survival (OS) and disease-specific survival (DSS). Correlation analyses were performed by the Spearman correlation test. Univariate and multivariate analyses were applied to establish a Cox proportional hazard regression model and a nomogram model. The time-dependent receiver operating characteristic (ROC) curves were generated to compare various survival factors. One-way ANOVA tests and Kruskal–Wallis tests were utilized to compare the difference between more than two groups. For experimental data, Student’s *t*-test was used to evaluate the differences between the two groups. Each experiment was performed three times, and all data were presented as the mean ± standard deviation (SD). Statistical significance was described as follows: ns, not significant; *p < 0.05; **p < 0.01; ***p < 0.001.

## Results

### RAB6B expression and its relationship with clinical parameters in hepatocellular carcinoma

**FIGURE 1:**
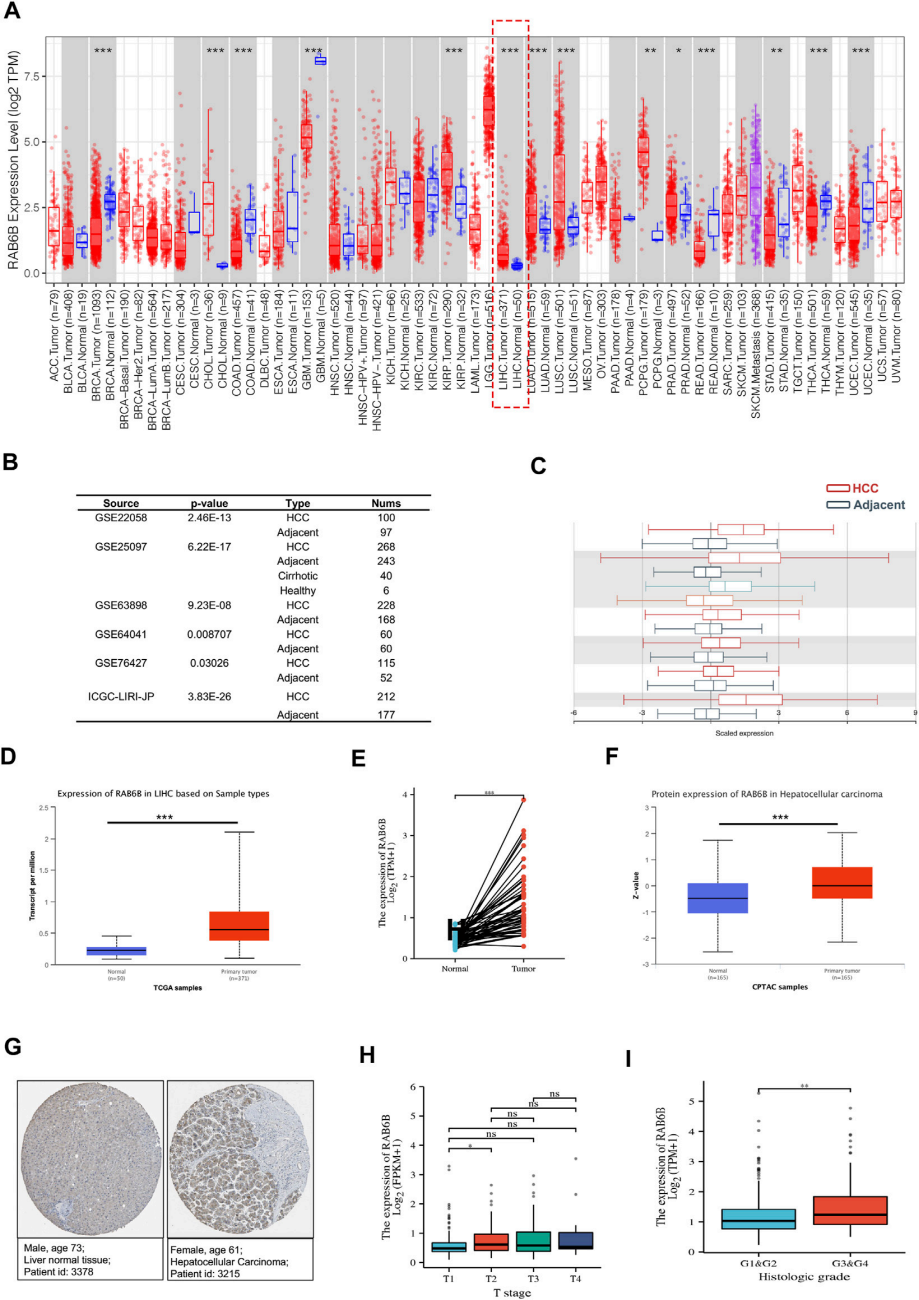
The expression level of RAB6B in HCC tissues. **(A)** RAB6B expression level in various tumor tissues and corresponding normal tissues in the TIMER2.0 database. RAB6B was upregulated in liver hepatocellular carcinoma (LIHC). *p < 0.05, **p < 0.01, and ***p < 0.001. **(B,C)** RAB6B was highly expressed in HCC tissues compared to the adjacent liver tissues in six HCC cohorts from the HCCDB database. **(D)** RAB6B mRNA expression level was increased in HCC tissues compared to normal liver tissues based on the UALCAN database. ***p < 0.001. **(E)** RAB6B expression was higher in 50 HCC tissues than in their paired adjacent normal liver tissues based on the TCGA database. ***p < 0.001. **(F)** RAB6B protein level in HCC tissues and normal tissues were obtained from the CPTAC dataset. ***p < 0.001. **(G)** Representative immunohistochemistry (IHC) images of RAB6B in HCC tissues (right image) and normal liver tissues (left image) from the HPA database. **(H)** Boxplot indicated that RAB6B expression was significantly associated with HCC patients’ T stage. *p < 0.05, ns = no significance. **(I)** Boxplot showed that RAB6B expression was correlated with histologic grade. **p < 0.01.

To explore the potential roles of RAB6B in HCC, we first evaluated the expression difference of RAB6B in HCC tissues and normal liver tissues. Analyzing TCGA data from the TIMER2.0 database, we found that the RAB6B expression was significantly elevated in HCC tissues relative to normal liver tissues (Figure 1A). Meanwhile, based on the HCCDB database analysis, the other five HCC GEO datasets (GSE22058, GSE25097, GSE63898, GSE64041, and GSE76427) and ICGC databases also showed that RAB6B was highly expressed in HCC tissues relative to adjacent tissues (Figures 1B,C). We also confirmed that the RAB6B mRNA expression level was upregulated in HCC tissues by using the UCLCAN database (Figure 1D). Furthermore, RAB6B expression was highly expressed in 50 paired HCC tissues (Figure 1E). In addition, using the CPTAC database, we found that the RAB6B protein expression level in HCC tissues was also higher than that in normal liver tissue (Figure 1F). Immunohistochemistry assays from the HPA database further verified that the RAB6B protein expression was significantly elevated in HCC tissues (Figure 1G).

Then, we explored the association between RAB6B expression and clinicopathological variables. As is shown in Table 1, RAB6B expression was higher in advanced T stages. Besides, RAB6B expression increased with the T classification and the histological grade (Figures 1H,I). Altogether, the above results indicated that the mRNA and protein levels of RAB6B were highly expressed in HCC tissues, and RAB6B expression was related to the T stages and grades in HCC patients.

**TABLE 1:** Correlations between the RAB6B expression and clinical characteristics of patients with HCC from the TCGA.

Characteristic	Total	High expression	Low expression	Pvalue
Age				0.4771
<=65	235 (62.83%)	121 (64.71%)	114 (60.96%)	
>65	138 (36.9%)	65 (34.76%)	73 (39.04%)	
Gender				0.1847
female	121 (32.35%)	67 (35.83%)	54 (28.88%)	
male	253 (67.65%)	120 (64.17%)	133 (71.12%)	
AFP				0.2226
high	117 (31.28%)	59 (31.55%)	58 (31.02%)	
normal	163 (43.58%)	69 (36.9%)	94 (50.27%)	
Stage				0.0337
stage I	173 (46.26%)	73 (39.04%)	100 (53.48%)	
stage II	87 (23.26%)	52 (27.81%)	35 (18.72%)	
stage III	85 (22.73%)	47 (25.13%)	38 (20.32%)	
stage IV	5 (1.34%)	3 (1.6%)	2 (1.07%)	
T				0.0211
T1	183 (48.93%)	77 (41.18%)	106 (56.68%)	
T2	95 (25.4%)	57 (30.48%)	38 (20.32%)	
T3	80 (21.39%)	45 (24.06%)	35 (18.72%)	
T4	13 (3.48%)	7 (3.74%)	6 (3.21%)	
M				1
M0	268 (71.66%)	138 (73.8%)	130 (69.52%)	
M1	4 (1.07%)	2 (1.07%)	2 (1.07%)	
N				0.67
N0	254 (67.91%)	131 (70.05%)	123 (65.78%)	
N1	4 (1.07%)	3 (1.6%)	1 (0.53%)	
Grade				0.089
G1	55 (14.71%)	23 (12.3%)	32 (17.11%)	
G2	178 (47.59%)	82 (43.85%)	96 (51.34%)	
G3	124 (33.16%)	71 (37.97%)	53 (28.34%)	
G4	12 (3.21%)	8 (4.28%)	4 (2.14%)	

### The prognostic and diagnostic value of RAB6B in patients with hepatocellular carcinoma

**FIGURE 2:**
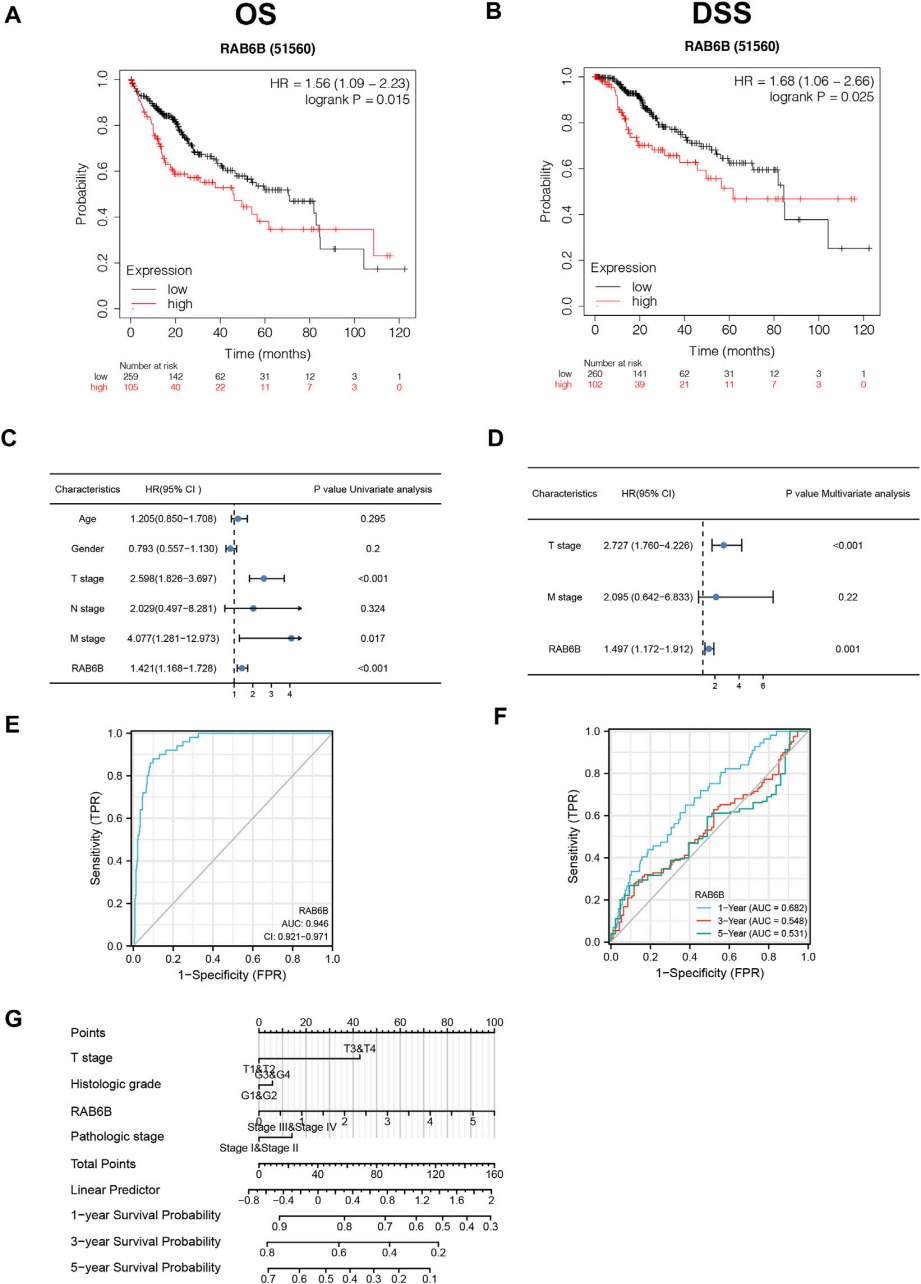
The prognostic and diagnostic value of RAB6B in patients with HCC. **(A,B)** Kaplan–Meier survival analysis showed that HCC patients with high RAB6B expression exhibited a shorter overall survival (OS) **(A)** and disease specific survival (DSS) **(B)** than those with low RAB6B expression. **(C)** Univariate COX analysis revealed that T stage, M stage, and RAB6B expression were significant factors affecting the survival rate of HCC patients. **(D)** Multivariate COX analysis showed that T stage and RAB6B expression were independent prognostic factors in HCC patients. **(E)** ROC curve was used to evaluate the diagnostic value of high RAB6B level for HCC patients in TCGA. **(F)** Time-dependent survival ROC curve analysis was used to predict 1-, 3- , and 5-years survival rates. **(G)** Nomogram integrating clinicopathologic features and RAB6B level was constructed to predict prognostic probabilities at 1-, 3-, and 5-years in the TCGA dataset.

Then, we wondered whether the high expression of RAB6B in HCC tissues affects the prognosis of HCC patients. Kaplan–Meier survival curves demonstrated that patients with high RAB6B expression tended to have poor overall survival (OS) (hazard ratio (HR) = 1.56, *p* = 0.015) and disease-specific survival (DSS) (HR = 1.68, *p* = 0.025) (Figures 2A,B). Meanwhile, univariate regression analysis revealed a relationship between prognosis with T stage, M stage, and RAB6B expression (Figure 2C). Furthermore, multivariate Cox regression analysis indicated that RAB6B expression (HR = 1.497, *p* = 0.001), and T stage (HR = 2.727, *p* < 0.001) were independent prognostic factors for HCC patients (Figure 2D).

In addition, a ROC curve analysis was conducted to evaluate the diagnostic performance of RAB6B in HCC. Area Under Curve (AUC) was found to be 0.946, which indicated that RAB6B expression was a highly reliable predictor (Figure 2E). The Time-dependent survival ROC curve of RAB6B was created to predict 1-, 3-, and 5-years survival rates. Relative to predicting 3-years (AUC = 0.548) and 5-years (AUC = 0.531) survival rates, the AUC value for predicting 1-year survival rate was 0.682, which showed a suitable predictive ability (Figure 2F). Furthermore, a nomogram model was performed according to the findings of multivariate Cox regression, which can be used to predict the survival probabilities at 1-, 3-, and 5-years for HCC patients (Figure 2G). Collectively, our results suggested that RAB6B may act as an independent prognosis factor and accurate diagnosis index in predicting OS among patients with HCC.

### RAB6B mutation landscape in hepatocellular carcinoma

**FIGURE 3:**
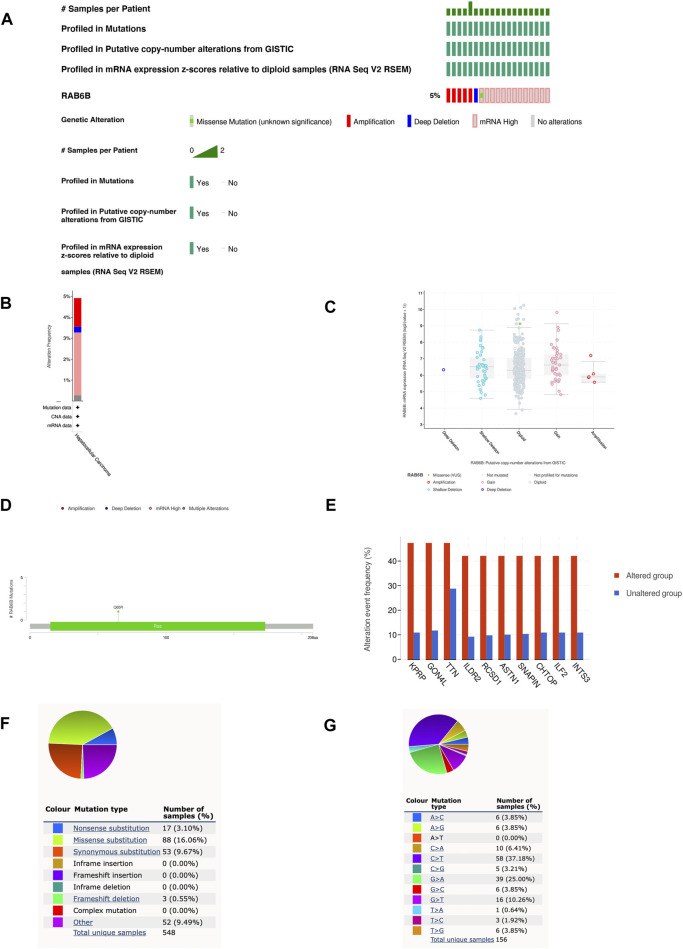
RAB6B mutation landscape in HCC. **(A)** OncoPrint summarized the genetic alterations in RAB6B based on LIHC (TCGA, Firehose Legacy, 379 samples) from the cBioPortal database. **(B)** RAB6B mutation frequency in HCC according to TCGA data. **(C)** The dot plot showed the correlation between RAB6B copy number and mRNA expression by cBioPortal. **(D)** Mutation diagram of RAB6B in HCC across protein domains. **(E)** The histogram showed the top 10 genes with the highest frequency in the genetic altered and unaltered group. **(F,G)** The pie chart showed the proportion of various mutation types of RAB6B in HCC based on the COSMIC database.

We then investigated the genetic alteration types and frequency of RAB6B in HCC based on the cBioPortal database. The TCGA-Firehose Legacy dataset, which contained 379 samples, was utilized for analysis. The alteration frequency of RAB6B was 5% in HCC, which included amplification, deep deletion, missense mutation, and mRNA high (Figure 3A). The histogram summarized the different types of genetic alterations of RAB6B in HCC samples (Figure 3B). Meanwhile, the relationship between different mutation types of RAB6B and mRNA expression was compared. HCC with RAB6B amplification had lower expression of mRNA relative to other type alterations of RAB6B (Figure 3C). The detailed mutation landscapes revealed that the Q65R missense mutation was the most frequent mutation site (Figure 3D). Additionally, we analyzed the top 10 significantly up-regulated genes in the gene-altered group relative to the unaltered group, including KPRP, GON4L, TTN, ILDR2, RCSD1, ASTN1, SNAPIN, CHTOP, ILF2, and INTS3 (Figure 3E). Using another database, COSMIC, we further assessed mutation types in RAB6B. As shown in the pie chart, missense substitutions accounted for the highest proportion at 16.06%, followed by synonymous substitutions at 9.67%, nonsense substitutions at 3.10%, and frameshift deletion at 0.55%, and other mutation types at 9.49% (Figure 3F). The top three substitution mutations were C > T (37.18%), G > A (25.00%), G > T (10.26%) (Figure 3G).

### RAB6B influence the infiltration of various tumor-associated immune cells in hepatocellular carcinoma

**FIGURE 4:**
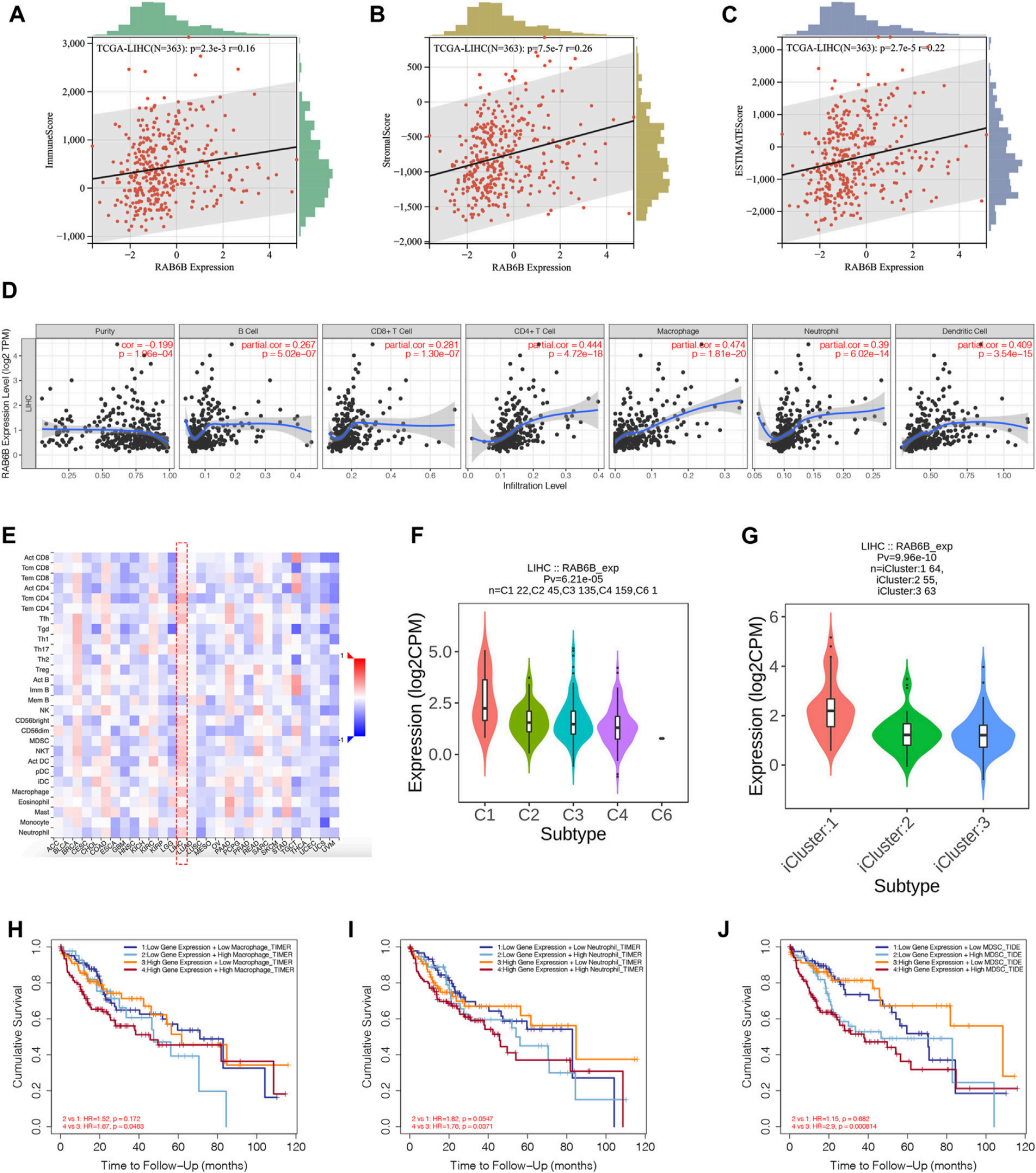
RAB6B expression correlated with tumor-infiltrating immune cells in HCC. **(A–C)** RAB6B expression was positively associated with the immune scores **(A)**, stromal scores **(B)**, and estimate scores **(C)** in HCC patients from the TCGA database. **(D)** Correlation between RAB6B expression and the tumor-infiltrating immune cells in HCC from the TIMER database. **(E)** Heatmap showed the relationship between RAB6B and various types of immune cells in HCC (red dashed rectangle) from the TISIDB database. **(F)** Correlation between RAB6B expression and immune subtypes in HCC from TISIDB database. C1 (wound healing); C2 (IFN-gamma dominant); C3 (inflammatory); C4 (lymphocyte depleted); C5 (immunologically quiet); C6 (TGF-b dominant). **(G)** Association between RAB6B expression and various molecular subtypes in HCC from TISIDB database. **(H–J)** Cumulative survival analysis of combinations of RAB6B expression and the abundance of macrophage cells **(H)**, neutrophil cells **(I)**, and MDSC **(J)** in HCC from the TIMER2.0 database. MDSC, myeloid-derived suppressor cells.

Accumulating studies have demonstrated that tumor progression is regulated by its surrounding immune microenvironment (Fu et al., 2019). Therefore, the relationship between RAB6B expression and tumor immune-infiltrating immune cells in HCC was explored. We first analyzed the association between RAB6B expression and immune score and stromal score of HCC patients, respectively, by using the ESTIMATE algorithm. The results showed that RAB6B expression was positively correlated with immune scores (r = 0.16, *p* = 2.3e-3), stromal scores (r = 0.26, *p* = 7.5e-7), as well as with estimate scores (r = 0.22, *p* = 2.7e-5) (Figures 4A–C). Additionally, the correlation between RAB6B expression and immune cell infiltration was further validated through TIMER database. We found that RAB6B expression was significantly positively correlated with infiltration level of B cells (r = 0.267, *p* = 5.02e-07), CD8^+^ T cells (r = 0.281, *p* = 1.30e-07), CD4^+^ T cells (r = 0.444, *p* = 4.72e-18), Macrophages (r = 0.474, *p* = 1.81e-20), Neutrophils (r = 0.39, *p* = 6.02e-14), and Dendritic cells (r = 0.409, *p* = 3.54e-15) but negatively correlated with tumor purity (r = −0.199, *p* = 1.96e-04) in HCC (Figure 4D). Consistently, using the TISIDB database, we found that RAB6B expression was positively correlated with the infiltrating abundance of most immune cells (Figure 4E), the corresponding r and *p* values were summarized in Table 2. Moreover, the GEPIA database was employed to confirm the relationship between the RAB6B expression and various immune cells markers in HCC, the r and *p* values were listed in Table 3.

**TABLE 2:** The correlation between RAB6B expression and tumor lymphocyte infiltration in HCC (TISIDB).

	LIHC	
	r	p
Activated CD8 T cell	0.097	6.04E-02
Central memory CD8 T cell	−0.025	6.31E-01
Effector memory CD8 T cell	0.077	1.37E-01
Activated CD4 T cell	0.132	**1.05E-02**
Central memory CD4T cell	0.319	**3.52E-10**
Effector memory CD4 T cell	0.152	**3.38E-03**
T follicular helper cell	0.258	**4.65E-07**
Gamma delta T cell	0.055	2.86E-01
Type 1 T helper cell	0.127	**1.40E-02**
Type 17 T helper cell	0.18	**5.00E-04**
Type 2 T helper cell	0.055	2.85E-01
Regulatory T cell	0.143	**5.68E-03**
Activated B cell	0.123	**1.73E-02**
Immature B cell	0.077	1.38E-01
Memory B cell	−0.21	**4.63E-05**
natural killer cell	−0.044	0.398
CD56bright natural killer cell	0.22	**1.98E-05**
CD56dim natural killer cell	0.153	**3.06E-03**
Myeloid derived suppressor cell	0.175	**6.75E-04**
Natural killer T cell	0.221	**1.71E-05**
Activated dendtritic cell	0.241	**2.74E-06**
Plasmacytoid dendtritic cell	0.154	**2.95E-03**
Immature dendtritic cell	0.031	0.553
Macrophage	0.241	**2.75E-06**
Eosinophil	0.027	0.597
Mast	0.252	**8.86E-07**
Monocyte	−0.046	0.371
Neutrophil	0.182	**4.26E-04**

Data are bolded to highlight that these data are statistically different..

**TABLE 3:** Correlation analysis between RAB6B expression and immune cell markers in HCC.

	Biomarker	Cor	p value
CD8+T	CD8A	0.21	1.00E-06
	CD8B	0.23	1.00E-07
Th1	T-bet (TBX21)	0.035	0.42
	STAT4	0.081	0.064
	STAT1	0.44	1.40E-26
	IFN-g (IFNG)	0.049	0.26
	TNF-a (TNF)	0.14	0.0016
Th2	GATA3	0.16	0.00015
	STAT5A	0.48	4.10E-32
	CCR3	0.35	2.10E-16
Tfh	BCL6	−0.012	0.78
	IL21	0.11	0.014
	CXCR5	0.31	2.10E-13
	ICOS	0.36	2.20E-17
Th17	STAT3	0.11	0.012
	IL17A	0.076	0.082
	IL-21R	0.33	7.00E-15
Treg	FOXP3	0.14	0.00094
	CCR8	0.47	3.60E-30
	TGFb (TGFB1)	0.56	2.00E-44
	IL2RA	0.4	3.80E-22
M1	INOS (NOS2)	0.41	2.10E-23
	IRF5	0.42	1.40E-24
	COX2 (PTGS2)	0.00018	1
M2	ARG1	−0.29	8.10E-12
	CD206 (MRC1)	−0.1	0.02
	CD115 (CSF1R)	0.32	6.50E-14
N	CD66b (CEACAM8)	−0.14	0.00084
	CD11b (ITGAM)	0.38	1.20E-19
	CCR7	0.2	5.00E-06
	FUT4	0.42	1.90E-24
NK	CD7	0.24	4.4-e08
	XCL1	0.15	7.00E-04
	KIR3DL1	−0.091	0.037
DC	HLA-DPB1	0.45	6.80E-28
	HLA-DQB1	0.35	2.80E-16
	HLA-DRA	0.46	2.10E-28
	HLA-DPA1	0.43	1.80E-25
	BDCA-1 (CD1C)	0.3	1.50E-12
	BDCA-4 (NRP1)	0.5	2.30E-34
	CD11c	0.32	2.00E-14
B	CD19	0.24	1.30E-08
	CD79A	0.13	0.0027
TAM	CCL2	0.22	2.40E-07
	CD68	0.32	2.10E-14
	IL10	0.1	0.021

According to the TISIDB database, HCC samples were divided into six immune subtypes: C1, wound healing; C2, IFN-gamma dominant; C3, inflammatory; C4, lymphocyte depleted; C5, immunologically quiet; and C6, TGF-b dominant. RAB6B expression was significantly correlated with the immune subtypes in HCC (*p* = 6.21e-05) (Figure 4F). Furthermore, RAB6B expression was also correlated with different molecular subtypes (*p* = 9.96e-10) (Figure 4G). Finally, we investigated the prognostic effect of RAB6B expression combined with different abundances of immune cell infiltration in HCC patients. Kaplan-Meier analysis showed that the survival rate of HCC patients with high expression of RAB6B and high infiltration of macrophages, neutrophils, and MDSCs was significantly reduced (Figures 4H–J). In conclusion, the above results indicated that RAB6B affected the infiltration of various immune cells in HCC and influenced the prognosis of HCC patients.

### RAB6B positively correlated with immunosuppressive microenvironment in hepatocellular carcinoma

**FIGURE 5:**
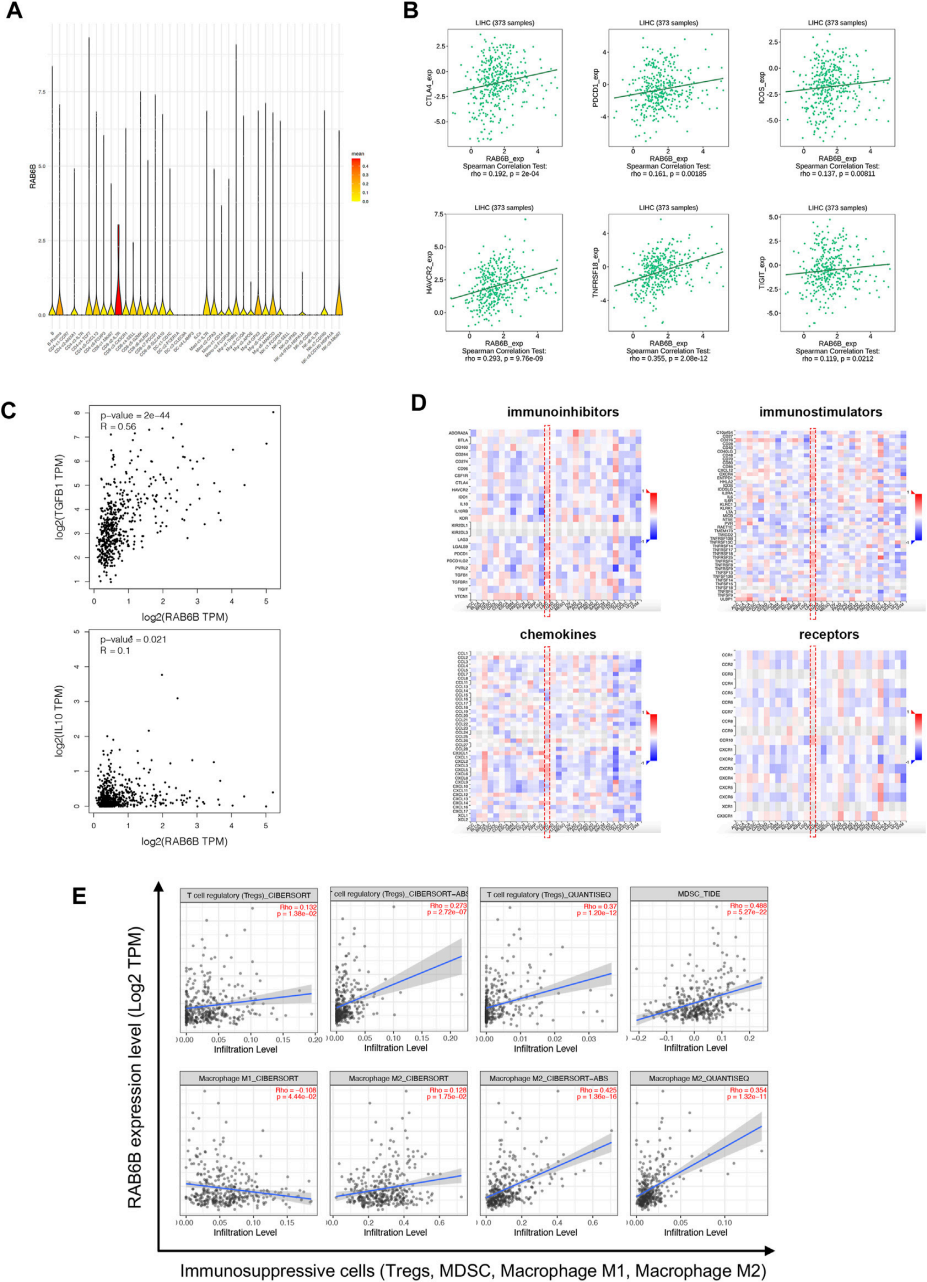
Relationship between RAB6B expression and immune-related molecules and cells. **(A)** RAB6B was mainly enriched in CD8+T cells according to single-cell RNA-sequencing results, which were analyzed by SMART-seq2. **(B)** Scatter plot showed that RAB6B expression was positively correlated with various immune checkpoint molecules, including CTLA4, PDCD1, ICOS, HAVCR2, TNFRSF18, and TIGIT. **(C)** RAB6B expression was positively correlated with TGFB1 and IL10 based on the GEPIA database. **(D)** The heatmap (red dashed rectangle) showed the correlation between RAB6B expression and various immunoinhibitors, immunostimulators, chemokines, and receptors in HCC from the TISIDB database. **(E)** RAB6B was positively correlated with Tregs, MDSC, and M2 macrophages infiltration, but negatively correlated with M1 macrophages infiltration by using the TIMER2.0 database. Tregs, regulatory T cells; MDSCs, myeloid-derived suppressor cells.

To further explore the effect of RAB6B on immune regulation in HCC, we analyzed single-cell RNA-sequencing data of HCC to investigate the relationship. The result showed that RAB6B was highly expressed on CD8^+^ T cells (Figure 5A). Since the HCC microenvironment is immunosuppressive, and the above results also indicated that RAB6B could also promote the infiltration of CD8^+^ T cells, we speculated that RAB6B might be involved in the induction of CD8^+^ T cells exhaustion. According to a recent study, exhausted CD8^+^ T cells could be divided into four stages (Beltra et al., 2020), and the correlation analysis was used to find that RAB6B expression was positively correlated with the main markers of each stage through the GEPIA database (Table 4). Moreover, RAB6B was also positively associated with the expression of immune checkpoint molecules, such as CTLA-4, PDCD1, ICOS, HAVCR2, TNFRSF18, and TIGIT (Figure 5B). Immunosuppressive cytokines, such as IL10 and TGF-β, are also involved in the regulation of CD8^+^ T cells exhaustion (Ejrnaes et al., 2006; Tinoco et al., 2009). Using the GEPIA database, we found that RAB6B was positively correlated with the expression of IL10 and TGF-b (Figure 5C). Besides, RAB6B was significantly associated with immunoinhibitors, immunostimulators, chemokines, and receptors in HCC (Figure 5D). Finally, we analyzed the association of RAB6B with various immunosuppressive lymphocytes in the TME using the TIMER2.0 database, including regulatory T cells (Tregs), myeloid-derived suppressor cells (MDSCs), and macrophages (M1 and M2). The analysis indicated that RAB6B was positively correlated with Tregs, MDSCs, and M2 macrophages, but negatively associated with M1 macrophages (Figure 5E). Taken together, RAB6B, which may be highly expressed in CD8^+^ T cells, participated in the regulation of CD8+T cells exhaustion by up-regulating the expression of immune checkpoint molecules, the secretion of immunosuppressive cytokines, and the recruitment of various immunosuppressive cells into HCC, thereby creating an immunosuppressive tumor microenvironment in HCC.

**TABLE 4:** The correlation between RAB6B expression and major genes of various stages of T cell exhaustion.

Stage	Gene	p value	R
Tex Prog1	CD28	6.20E-17	0.35
	CXCR5	2.10E-13	0.31
	ICOS	2.20E-17	0.36
	MYB	2.10E-19	0.38
	OAS1	2.80E-02	0.095
	SELL	8.50E-09	0.25
	STAT1	1.40E-26	0.44
	TCF7	9.50E-08	0.23
Tex Prog2	ALCAM	3.10E-24	0.42
	ANXA2	8.10E-59	0.63
	ITGB7	1.20E-03	0.14
	MKI67	1.10E-44	0.56
Tex Int	CX3CR1	1.20E-07	0.23
	GZMA	1.10E-06	0.21
	GZMB	8.60E-02	0.075
	KLRK1	1.20E-01	−0.068
	PRDM1	4.60E-26	0.44
	PRF1	4.90E-01	0.03
	ZEB2	1.30E-07	0.23
Tex Term	CD38	5.70E-04	0.15
	CD101	1.20E-26	0.44
	ENTPD1	2.20E-80	0.7

### RAB6B co-expression networks in hepatocellular carcinoma

**FIGURE 6:**
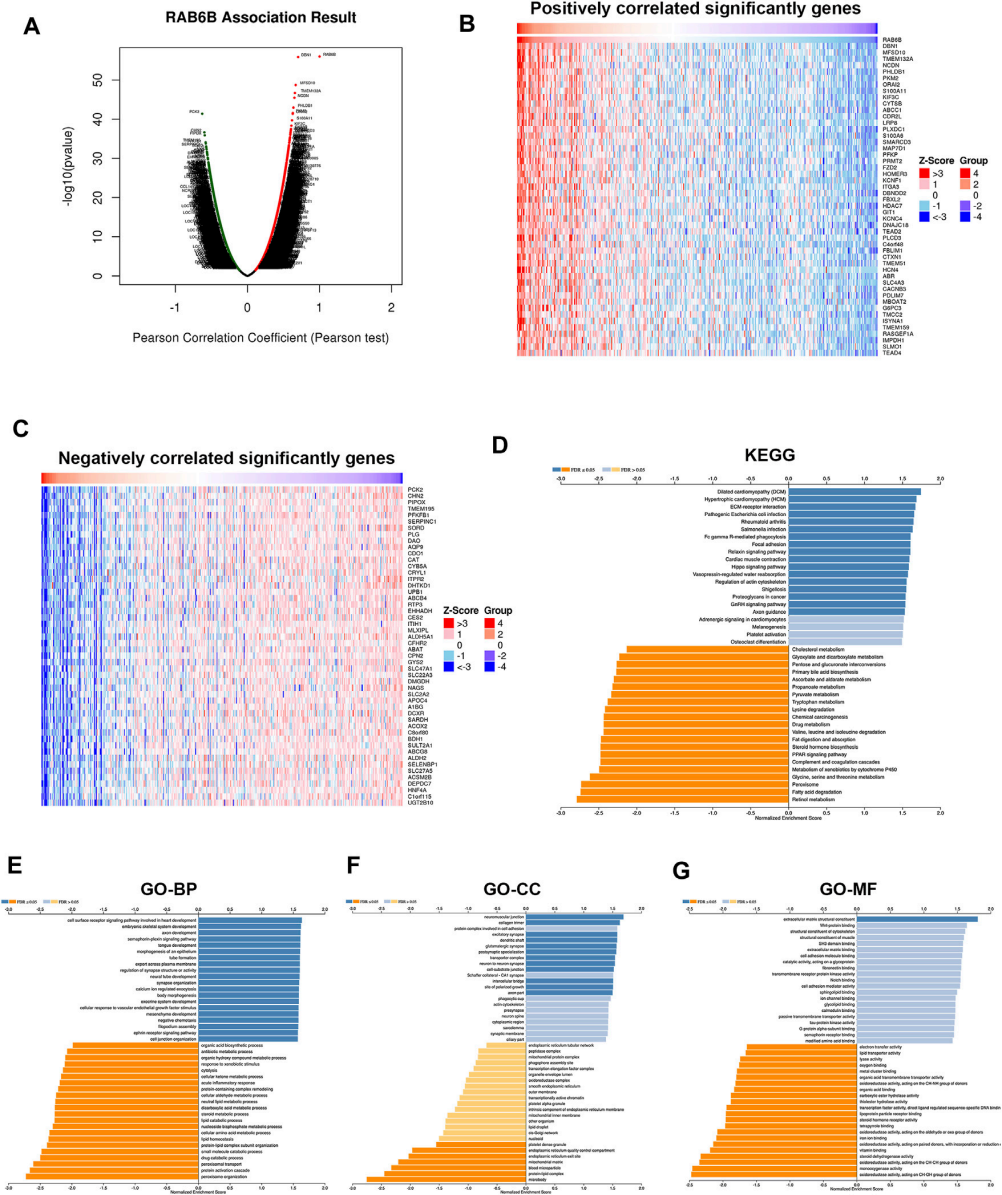
Functional enrichment analysis of RAB6B-related genes. **(A)** The Volcano plot showed RAB6B co-expressed gene profile in HCC bythe LinkedOmics database. **(B,C)** Heatmap showed 50 positively **(B)** and 50 negatively **(C)** correlated genes with RAB6B expression in HCC. **(D)** KEGG analysis of RAB6B co-expressed genes in HCC. KEGG, Kyoto Encyclopedia of Genes and Genomes. **(E–G)** Various types of GO analysis of RAB6B co-expressed genes in HCC. GO, Gene Ontology; BP, Biological Process; CC, Cellular Component; MF, Molecular Function.

To investigate the biological role of RAB6B, the co-expression pattern of RAB6B was explored in TCGA- LIHC cohort through LinkedOmics. The results showed that 9,100 genes were positively correlated with RAB6B, while 3451 genes were negatively correlated with RAB6B (Figure 6A). The top 50 positively and negatively correlated genes were presented in heat maps (Figures 6B,C). KEGG pathway analysis indicated that enrichment mainly in the extracellular matrix (ECM)-receptor interaction, Fc-gamma R-mediated phagocytosis, Focal adhesion, and Hippo signaling pathway (Figure 6D). The results of GO enrichment analysis suggested that RAB6B co-expressed genes involved mainly in calcium ion regulated exocytosis, collagen trimer, and extracellular matrix structural constituent (Figures 6E–G). All these results indicated that the RAB6B co-expression network may influence the tumor stroma in the TME of HCC.

### RAB6B is correlated with cancer-associated fibroblasts in hepatocellular carcinoma

**FIGURE 7:**
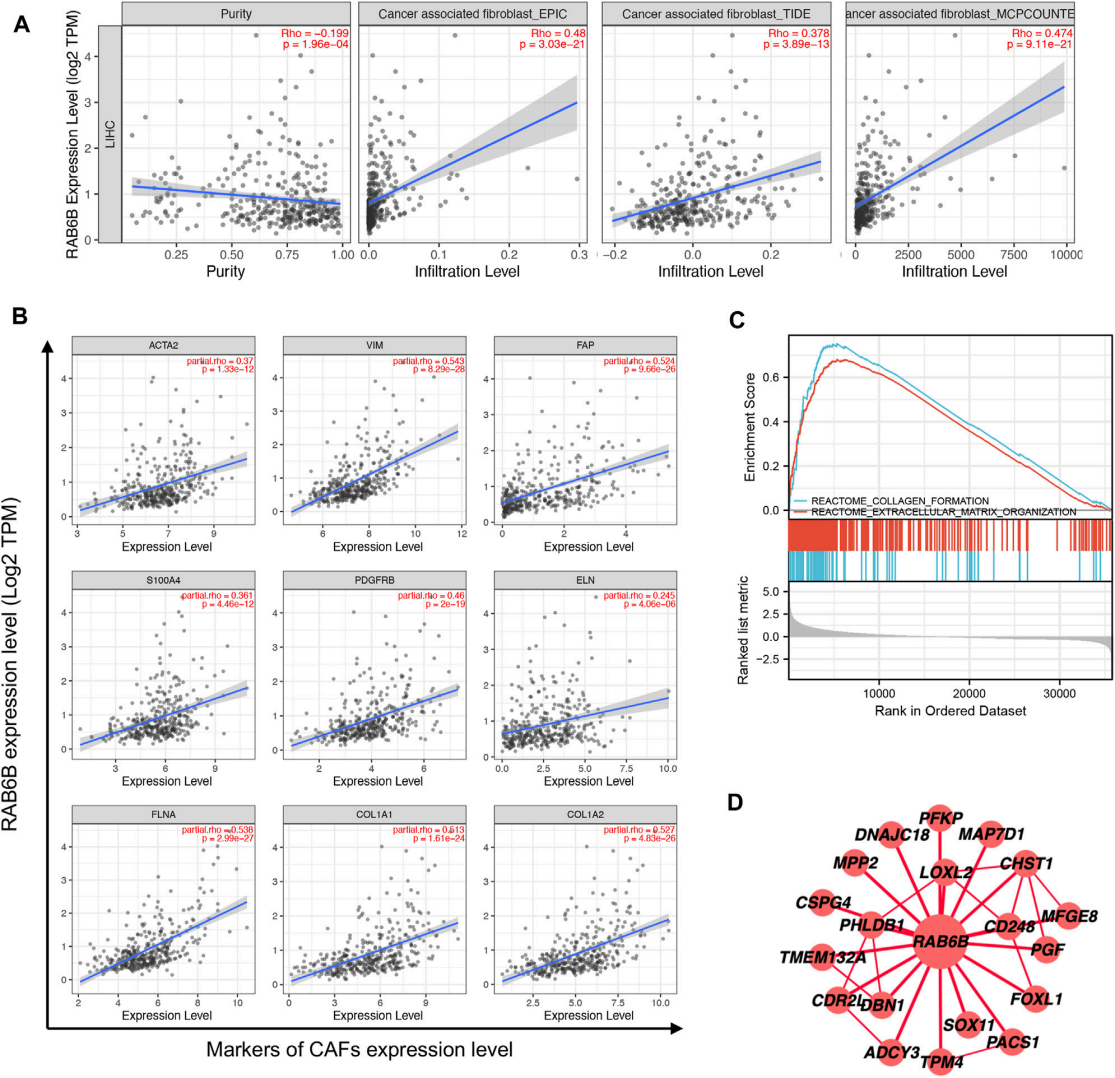
Correlation between the infiltration of CAFs and RAB6B expression in HCC. **(A)** RAB6B expression was positively correlated with the infiltration of CAFs in HCC based on the TIMER2.0 database. **(B)** RAB6B expression was positively associated with the main markers of CAFs, including ACTA2, VIM, FAP, S100A4, and PDGFRB, and was positively correlated with the expression of collagen-related proteins, including ELN, FLNA, COL1A1, and COL1A2. CAFs, cancer-associated fibroblasts. **(C)** The enrichment results of GSEA showed that upregulated RAB6B was positively correlated with “collagen formation” and “extracellular matrix organization.” **(D)** The RAB6B PPI network was performed from the HCCDB database. PPI, protein-protein interaction.

The above results showed a positive correlation between RAB6B expression and stromal score, concerning cancer-associated fibroblasts (CAFs) were the main components of tumor stroma (Yin et al., 2019), so we explored the relationship between RAB6B and tumor-infiltrating CAFs. Through the TIMER2.0 database, the results showed that RAB6B expression was positively correlated with the abundance of CAFs infiltration in HCC by using different algorithms, including EPIC, TIDE, and MCPCOUNTER (Figure 7A). Then, we analyzed the relationship between the main markers of CAFs and RAB6B expression, the results revealed that RAB6B was positively correlated with ACTA2 (r = 0.37, *p* = 1.33e-12), Vimentin (r = 0.543, *p* = 8.29e-28), FAP (r = 0.524, *p* = 9.66e-26), S100A4 (r = 0.361, *p* = 4.46e-12), PDGFRB (r = 0.46, p = 2e-19). Moreover, RAB6B was also positively associated with collagen-related genes, including ELN (r = 0.245, *p* = 4.06e-06), FLNA (r = 0.538, *p* = 2.99e-27), COL1A1 (r = 0.513, *p* = 1.61e-24), COL1A2 (r = 0.527, *p* = 4.83e-26) (Figure 7B). We also performed GSEA to explore the potential biological process of RAB6B expression in HCC. The top 10 pathways significantly positively and negatively associated with RAB6B expression in HCC were presented in Supplementary Figure S1. The GSEA results confirmed that collagen formation and extracellular matrix organization were significantly gathered in the high-RAB6B expression group (Figure 7C). Finally, the co-expressed proteins network of RAB6B was investigated by using the HCCDB database, the results showed that RAB6B may participate in regulating the expression of LOXL2, CD248, and MPP2 et al., thereby affecting the homeostasis of ECM (Figure 7D). Collectively, these findings indicated that RAB6B may promote the infiltration of CAFs and ECM remolding, thereby reshaping the TME of HCC.

### Correlation between RAB6B expression and drug response

**FIGURE 8:**
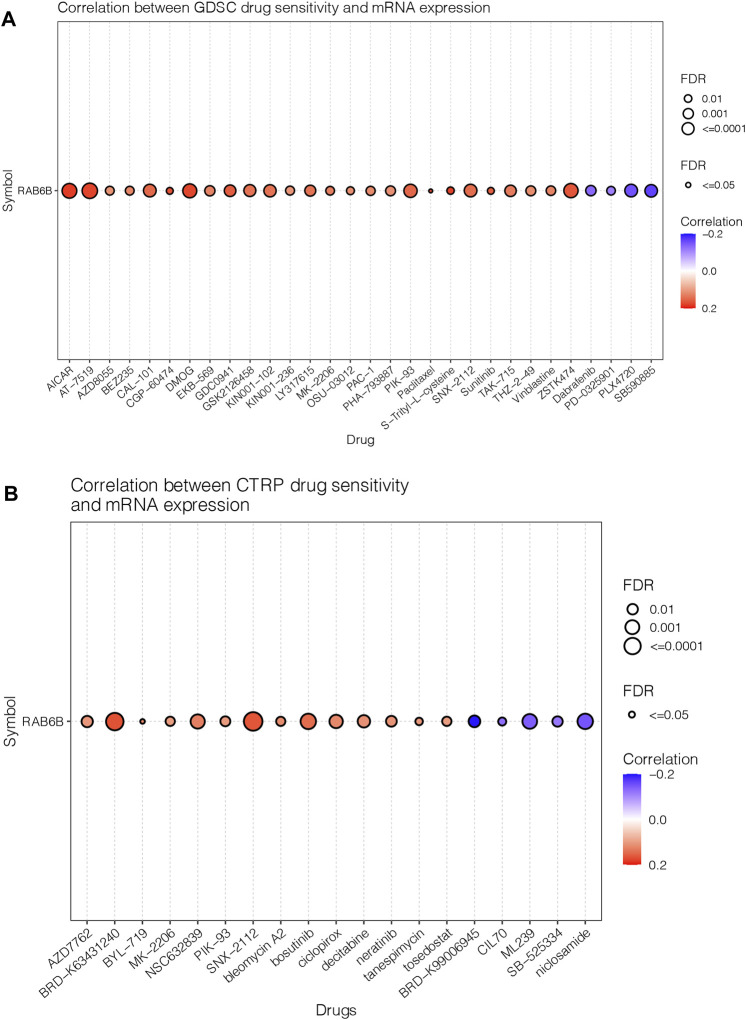
Correlation between RAB6B expression and drug response. **(A)** Dot plot summarized the relationship between RAB6B mRNA expression and the sensitivity of GDSC drugs (top 30) in pan-cancer. GDSC, Genomics of Drug Sensitivity in Cancer. **(B)** RAB6B mRNA expression and the sensitivity of CTRP drugs (top 30) in pan-cancer. CTRP, Cancer therapeutics Response Portal.

Next, we wanted to explore the responsiveness of RAB6B to chemotherapy, the RAB6B mRNA expression and drug sensitivity were integrated through GDSC and CTRP databases. The results showed that in the GDSC database, RAB6B was positively correlated to IC50 of AICAR, AT-7519, AZD8055, BEZ235, CAL-101, CGP-60474, DMOG, EKB-569, GDC0941, GSK2126458, KIN001-102, KIN001-236, LY317615, MK-2206, OSU-03012, PAC-1, Paclitaxel, PHA-793887, PIK-93, SNX-2112, S-Trityl-L-cysteine, Sunitinib, TAK-715, and THZ-2–49, Vinblastine, ZSTK474, while was negatively associated with Dabrafenib, PD-0325901, PLX4720, SB590885 (Figure 8A). Based on the CTRP database, RAB6B displayed a positive relationship to IC50 of AZD7762, BRD-K63431240, BYL-719, MK-2206, NSC632839, PIK-93, SNX-2112, bleomycin A2, bosutinib, ciclopirox, decitabine, neratinib, tanespimycin, tosedostat but was negatively correlated to IC50 of BRD-K99006945, CIL70, ML239, SB-525334, niclosamide (Figure 8B). The specific correlation coefficients and targets with drugs of GDSC and CTRP were summarized respectively in Tables 5, 6. Taken together, most of these drugs were positively associated with RAB6B expression based on the IC50.

**TABLE 5:** Correlation between GDSC drug sensitivity and RAB6B mRNA expression.

Gene Symbol	Drug	Cor	Fdr	Target	Target pathway
RAB6B	AICAR	0.18188208	1.108421522E-06	AMPK agonist	Metabolism
	AT-7519	0.176693098	3.240025473E-07	CDK1, CDK2, CDK4, CDK6, CDK9	Cell cycle
	AZD8055	0.111224671	0.003751073	MTORC1, MTORC2	PI3K/MTOR signaling
	BEZ235	0.122117559	0.003652679	PI3K (class 1), MTORC1, MTORC2	PI3K/MTOR signaling
	CAL-101	0.148743384	3.2573E-05	PI3Kdelta	PI3K/MTOR signaling
	CGP-60474	0.167396186	0.01465139	CDK1,CDK2,CDK5,CDK7,CDK9, PKC	Cell cycle
	DMOG	0.175839264	4.559166636E-06	HIF-PH	Metabolism
	EKB-569	0.124046127	0.000954349	EGFR	EGFR signaling
	GDC0941	0.15762539	0.000172875	PI3K (class 1)	PI3K/MTOR signaling
	GSK2126458	0.139609616	0.000101231	PI3K (class 1), MTORC1, MTORC2	PI3K/MTOR signaling
	KIN001-102	0.138786462	6.7699E-05	AKT1, AKT2, AKT3	PI3K/MTOR signaling
	KIN001-236	0.106615078	0.003261655	Angiopoietin-1 receptor	RTK signaling
	LY317615	0.138465471	0.000371265	PKCB	Other, kinases
	MK-2206	0.129034024	0.004032457	AKT1, AKT2	PI3K/MTOR signaling
	OSU-03012	0.114852773	0.007637659	PDK1 (PDPK1)	Metabolism
	PAC-1	0.117320981	0.002803922	Procaspase-3, Procaspase-7	Apoptosis regulation
	Paclitaxel	0.171294461	0.032168205	Microtubule stabiliser	Mitosis
	PHA-793887	0.111571589	0.001409063	CDK2, CDK7, CDK5	Cell cycle
	PIK-93	0.150308436	1.4104E-05	PI3Kgamma	PI3K/MTOR signaling
	SNX-2112	0.145501484	3.6887E-05	HSP90	Protein stability and degradation
	S-Trityl-L-cysteine	0.178037205	0.010851251	KIF11	Mitosis
	Sunitinib	0.157548895	0.01407095	PDGFR, KIT, VEGFR, FLT3, RET, CSF1R	RTK signaling
	TAK-715	0.131352173	0.000203855	p38alpha, p38beta	JNK and p38 signaling
	THZ-2-49	0.116025378	0.001302119	CDK9	Cell cycle
	Vinblastine	0.124267764	0.00192142	Microtubule destabiliser	Mitosis
	ZSTK474	0.164483126	2.662594419E-06	PI3K (class 1)	PI3K/MTOR signaling
	Dabrafenib	-0.129933621	0.000794279	BRAF	ERK MAPK signaling
	PD-0325901	-0.109929629	0.00436616	MEK1, MEK2	ERK MAPK signaling
	PLX4720	-0.151194872	3.6483E-05	BRAF	ERK MAPK signaling
	SB590885	-0.164353004	4.4315E-05	BRAF	ERK MAPK signaling

**TABLE 6:** Correlation between CTRP drug sensitivity and RAB6B mRNA expression.

Gene symbol	Drug	Cor	Fdr	Target
RAB6B	AZD7762	0.105141148	0.006363712	inhibitor of checkpoint kinases 1 and 2
	BRD-K63431240	0.167889073	3.1119E-05	product of diversity oriented synthesis
	BYL-719	0.118366783	0.045070757	inhibitor of PI3K catalytic subunit alpha
	MK-2206	0.101297324	0.017278249	inhibitor of AKT1
	NSC632839	0.127547938	0.00082705	inhibitor of USP2, USP7 和 SENP2
	PIK-93	0.101684596	0.013107972	inhibitor of PI3K catalytic subunit gamma
	SNX-2112	0.16503565	8.467415545E-06	inhibitor of HSP90alpha and HSP90beta
	bleomycin A2	0.115929242	0.018053065	inducer of DNA damage
	bosutinib	0.145373518	0.000265119	inhibitor of SRC and ABL1
	ciclopirox	0.119001893	0.001514873	inhibitor of the iron-dependent enzyme ribonucleotide reductase
	decitabine	0.111592642	0.003158139	inhibitor of DNA methyltransferase
	neratinib	0.109379465	0.008598225	inhibitor of EGFR and HER2
	tanespimycin	0.105157895	0.031245529	inhibitor of HSP90
	tosedostat	0.10017112	0.017191426	inhibitor of ANPEP;LAP3;NPEPPS
	BRD-K99006945	−0.194235972	0.004936331	inhibitor of TP53
	CIL70	−0.125656697	0.026917134	Unknown
	ML239	−0.139809968	0.000518539	inhibitor of breast cancer stem cell proliferation
	SB-525334	−0.117023472	0.010787288	inhibitor of the transforming growth factor beta type 1 receptor
	niclosamide	−0.148197981	0.000276337	inhibitor of STAT3 signaling

### Knockdown of RAB6B inhibits hepatocellular carcinoma cells proliferation, promotes apoptosis, and enhances the sensitivity to cisplatin

**FIGURE 9:**
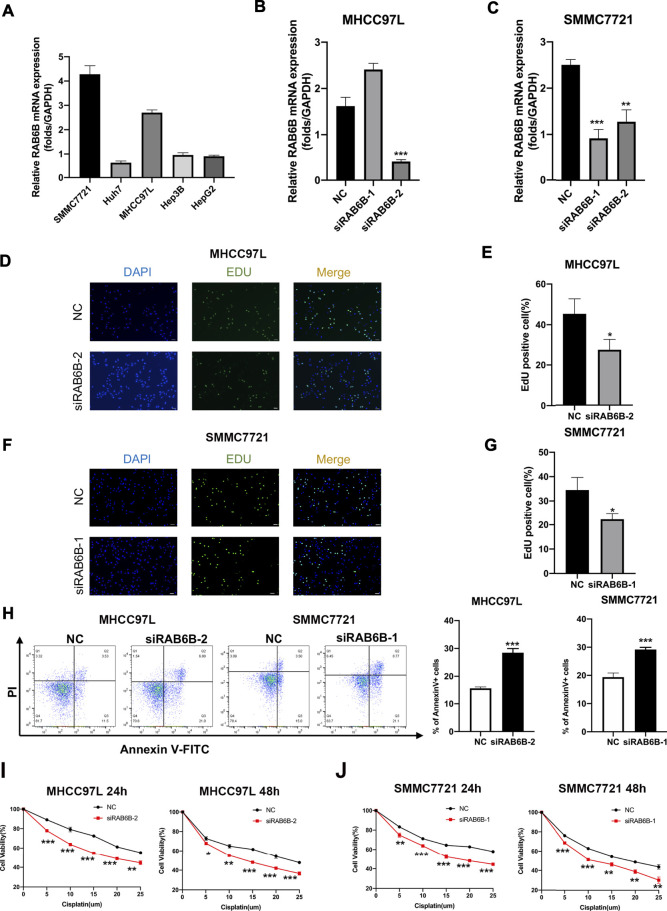
Effects of RAB6B knockdown on cell proliferation, apoptosis, and drug sensitivity in HCC cells. **(A)** The qRT-PCR result showed the RAB6B mRNA Expression in various HCC cell lines. **(B)** The qRT-PCR result showed the knockdown efficiency of RAB6B after transfection of MHCC97L cells with two different siRNAs. **(C)** Knockdown efficiency of RAB6B after transfection of SMMC7721 cells with two different siRNAs. **(D,E)** The effect of RAB6B knockdown on cell proliferation in MHCC97L cells was performed by EdU assay. Scale bar, 20um. **(F,G)** The effect of RAB6B knockdown on cell proliferation in SMMC7721 cells was performed by EdU assay. Scale bar, 20 µm. **(H)** The effect of RAB6B knockdown on cell apoptosis in MHCC97L and SMMC7721 cells performed by flow cytometry. **(I,J)** Gradient doses of cisplatin were used to treat MHCC97L cells **(I)** or SMMC7721 cells **(J)** for 24 and 48 h, and the cell viability was measured by MTT assay. All above data were presented as the mean ± SD, *n* = 3. NC, negative control. *p < 0.05; **p < 0.01; ***p < 0.001.

Based on the above bioinformatics analysis results, the effect of RAB6B on the biological behavior of HCC was invalidated *in vitro*. The RAB6B mRNA expression in five HCC cell lines was compared, and the results showed that SMMC772 and MHCC97L had relatively higher RAB6B expression levels (Figure 9A). Therefore, RAB6B was knockdown in MHCC97L and SMMC7721 cells by transfection with siRNAs for subsequent experiments *in vitro*. qRT-PCR was performed to examine the knockdown efficiency of various siRNAs. The results showed that siRAB6B-2 significantly inhibited the RAB6B mRNA expression level in MHCC97L cells, while both siRAB6B-1 and siRAB6B-2 decreased the RAB6B mRNA expression in SMMC7721 cells (Figures 9B,C). To avoid off-target effects of siRNA, siRAB6B-2 and siRAB6B-1 were used to transfect MHCC97 L and SMMC7721 cells, respectively.

The EdU assay was performed to investigate the role of RAB6B knockdown on HCC cell proliferation, and the results revealed that the proliferation of MHCC97L and SMMC7721 cells was significantly decreased after RAB6B inhibition (Figures 9D–G). Moreover, after the knockdown of RAB6B, the percentage of apoptotic MHCC97L and SMMC7721 cells was significantly increased by using flow cytometry (Figure 9H). Finally, MHCC97L and SMMC7721 cells were treated with gradient doses of cisplatin for 24 and 48 h, and the results found that knockdown of RAB6B significantly reduced the resistance to cisplatin by MTT assay (Figures 9I,J). Overall, these results suggested that RAB6B may be involved in the regulation of HCC cell proliferation, apoptosis, and sensitivity to cisplatin.

## Discussion

Rab GTPases are a highly conserved family of regulatory genes involved in vesicular transport (Li and Marlin, 2015). Each Rab GTPases localizes in a specific subcellular structure and exerts its respective membrane trafficking functions (Stenmark, 2009). Previous studies have reported that several Rab GTPases are involved in HCC progression, including modulating proliferation, migration, invasion, and metastasis (Yang et al., 2021). RAB6B, an isoform of RAB6, is mainly located in the Golgi apparatus and plays a role in Golgi-to-ER retrograde transport (Wanschers et al., 2007). Prior study has shown that high RAB6B expression promoted cell proliferation and the cell cycle G1/S phase transition through AKT/JNK signaling pathways in gastric cancer (Zhao et al., 2020). By contrast, RAB6B was low expressed in pancreatic and colorectal cancers, and correlated with poorer prognosis in patients (Anand et al., 2020; Jiang et al., 2022). These results suggested that RAB6B may play specific roles in different TME. Besides, few studies have reported the role of the Rab GTPases in the tumor immune microenvironment (TIME) of HCC. Therefore, we here mainly explored the potential functions of RAB6B in HCC based on public databases, including its role in tumor-associated immune cells infiltration and tumor stroma remodeling.

In this study, we found that RAB6B mRNA and protein expression levels were significantly upregulated in HCC compared to normal liver tissues across various public databases. Clinical association analyses demonstrated that increased RAB6B expression was correlated with higher T stage, and histological grade. Moreover, HCC patients with high RAB6B expression showed worse OS and DSS based on Kaplan–Meier analysis. Univariate and multivariate Cox regression analyses further revealed that high RAB6B expression was an independent risk factor to predict poor OS for HCC patients. Additionally, ROC curve analysis showed that RAB6B has excellent diagnostic value in HCC.

Uncontrolled proliferation of tumor cells caused by key driver gene mutations often leads to tumorigenesis (El Tekle et al., 2021). Moreover, recent studies have found that there exist context-dependent genetic mutations, where a specific mutation plays a major role in a certain tumor type (Hoadley et al., 2018). Therefore, we explored the proportion and types of RAB6B mutations in liver cancer. The percentage of RAB6B genetical alterations reached 5%, of which missense substitutions accounted for the highest proportion of 16.06%. Meanwhile, we analyzed the top 10 genes that were significantly upregulated in the genetically altered group, including KPRP, GON4L, TTN, ILDR2, RCSD1, ASTN1, SNAPIN, CHTOP, ILF2, and INTS3. Among them, GON4L is a transcriptional regulator gene that has been reported to drive tumor growth through the YY1-androgen receptor-CD24 pathway (Agarwal et al., 2016). ILF2 (Interleukin enhancer binding factor 2), a transcription factor, is upregulated in HCC and can promote HCC tumorigenesis *in vivo* and *in vitro* (Cheng et al., 2016). INTS3 (integrator complex subunit 3), is found to be significantly overexpressed in HCC tissues and may be involved in HCC development (Inagaki et al., 2008). These results suggested that the genetic alterations of RAB6B in HCC may potentially activate the expression of the above-mentioned oncogenes or pathways to promote the progression of HCC.

According to the “seed and soil” theory, tumor cells act as seeds and continuously interact with the surrounding microenvironment to jointly promote tumor progression (Quail and Joyce, 2013). Targeting cancer cells alone usually cannot acquire a satisfactory effect. Recently, accumulating studies have focused on the research of TME. The TME is composed of various noncancer cellular components surrounding tumor cells, including tumor-infiltrating lymphocytes (TILs), CAFs, ECM, and endothelial cells (Chen and Song, 2019). Therefore, we explored the effect of RAB6B on the TME based on public databases. Our results revealed that RAB6B expression was positively correlated with immune scores and stromal scores by using the ESTIMATE algorithm. Meanwhile, RAB6B expression was related to the infiltrating abundance of various tumor-associated immune cells in HCC, including B cells, CD8^+^ T cells, CD4^+^ T cells, macrophages, neutrophils, and DCs. Furthermore, the survival analysis in combination with gene expression and immune cells infiltration abundance showed that HCC patients with high RAB6B expression and macrophages or neutrophils or MDSC cells infiltration tend to have a worse prognosis in HCC.

TILs can be divided into anti-tumor immune cells and immunosuppressive cells. The former include CD8^+^ T cells, natural killer (NK) cells, DC cells, type 1-polarized macrophages (M1) and the latter includes myeloid-derived suppressor cells (MDSCs), regulatory T cells (Tregs), and type 2-polarized macrophages (M2) (Lu et al., 2019). We analyzed the single-cell sequencing data of HCC and found that RAB6B was significantly overexpressed in CD8^+^ T cells, considering the TME of HCC is an immunosuppressive state, we speculated that RAB6B may be involved in the regulation of CD8^+^ T cell exhaustion in HCC. According to a newly published study, exhausted CD8^+^ T cells can be divided into four stages, namely T cell exhaustion progenitors 1 (Tex Prog1), T cell exhaustion progenitors 2 (TexProg2), T cell exhaustion intermediate (TexInt), and T cell exhaustion terminally (Tex Term) (Beltra et al., 2020). By using the GEPIA database, we found that RAB6B was positively correlated with each exhausted stage of CD8+T cells. Previous studies have shown that the upregulation of immune checkpoint molecules can regulate T cells exhaustion, such as CTLA-4, PDCD1, ICOS, HAVCR2, TNFRSF18, and TIGIT (Wherry, 2011). Our results suggested that RAB6B was also positively associated with the aforementioned immune checkpoint molecules. In addition, we found that RAB6B may promote the production of immunosuppressive cytokines, such as IL10 and TGF-β, which have been reported to inhibit T cells function in TME (Ejrnaes et al., 2006; Tinoco et al., 2009). Finally, based on the TIMER database, our results revealed that RAB6B was positively correlated with immunosuppressive cells, such as MDSCs, Tregs, and M2 macrophages, but negatively correlates with M1 macrophages. All these findings indicated that RAB6B may induce the exhaustion of CD8^+^ T cells, recruiting various immunosuppressive cells and cytokines into the TME, thereby promoting the formation of the immunosuppressive microenvironment of HCC.

Next, to further investigate the role of RAB6B in the progression of HCC, we identified genes co-expressed with RAB6B and performed functional enrichment analysis based on the group of differential co-expressed genes. The KEGG results revealed that the enrichment was primarily associated with ECM-receptor interaction, immune-related diseases (pathogenic *Escherichia coli* infection, *Salmonella* infection, Rheumatoid arthritis), Fc-gamma R-mediated phagocytosis, Focal adhesion, Hippo signaling pathway, regulation of actin cytoskeleton. GO analysis displayed that RAB6B expression was mainly involved in collagen trimer, cell adhesion, and ECM structural constituent. The functional enrichment results showed that RAB6B may be associated with ECM remodeling in the TME, so we explored the regulatory role of RAB6B on tumor stroma. Increasing studies have shown that CAFs, as the main components of tumor stroma, promote tumor progression by interacting with various cell components in the TME (Jia et al., 2021). ECM is a noncellular component of TME and is mainly produced by CAFs (Chaudhuri et al., 2014). During the tumor development, ECM continuously undergoes cross-linking, rearrangement, and degradation, thereby influencing tumor invasion, drug resistance, and metastasis (Elosegui-Artola et al., 2016). Using the TIMER2.0 database, different algorithms were used to find that RAB6B expression was positively correlated with the infiltration levels of CAFs in HCC. Previous studies have reported that α-smooth muscle actin (α-SMA), fibroblast activation protein (FAP), vimentin, platelet-derived growth factor (PDGF) receptor (PDGFR)-β, and fibroblast-specific protein 1 (FSP-1) can be served as the markers to identify CAFs. Our correlation analysis suggested that RAB6B was positively correlated with the genes encoding these proteins in HCC. Meanwhile, we also found that RAB6B was positively correlated with ECM-related genes, such as ELN, FLNA, COL1A1, and COL1A2. Through PPI network construction, several proteins, such as LOXL2, CD248, MPP2 et, al. Were found to be co-expressed with RAB6B in HCC. Lysyl oxidase-like 2 (LOXL-2), a collagen-modifying enzyme, has been found to increase the stiffness of tumor tissue by modifying the ECM components in the TME in HCC, thereby promoting intrahepatic metastasis (Wong et al., 2014). CD248, also known as Endosialin, is a transmembrane glycoprotein, that has been reported to be expressed mainly in CAFs in HCC, involved in the recruitment and M2 polarization of macrophages to promote HCC progression (Yang et al., 2020). In addition, Tropomyosin4 (TPM4), a member of actin-binding proteins, was also found to be highly expressed in HCC and correlated with poor prognosis in HCC patients (Li et al., 2021). These potentially interacting proteins suggested that RAB6B may be involved in remodeling the TME in HCC.

Most HCC patients are diagnosed at an advanced stage, and the treatment options are limited, usually targeted therapy, radiotherapy, and chemotherapy (Chen et al., 2019). Therefore, the potential RAB6B-responsive drugs based on GDSC and CTRP databases were mined. We found RAB6B positively correlated with IC50 of most drugs, most of which targeted PI3K/mTOR signaling. Recently, immunotherapy has emerged as a promising therapy in the treatment of several solid tumors, and various immune checkpoint molecules, such as PD-1/PD-L1 and CTLA-4, have been found to play an imperial role in tumor immune escape (Chang et al., 2017). Furthermore, the application of immune checkpoint inhibitors (ICIs) can enhance the immune response of tumors and inhibit tumor progression. Since we found that RAB6B was highly correlated with the expression of various immune checkpoint molecules and promoted the formation of the immunosuppressive microenvironment. Thus, the combination of ICIs and chemotherapeutics targeting RAB6B can provide a potent rationale for the treatment of HCC.

Based on the above bioinformatics findings, a series of functional assays were carried out *in vitro* by downregulating the RAB6B expression. The results showed that RAB6B knockdown inhibited cell proliferation and promoted apoptosis of HCC cells *in vitro*. In addition, inhibition of RAB6B enhanced the sensitivity to chemotherapeutic drugs such as cisplatin. However, our study also has some limitations, such as our findings were based on the analysis of public databases data, and the role of RAB6B in immune infiltration in TME needs to be examined *in vitro* and *in vivo*, meanwhile, the mechanism of RAB6B on HCC needs to be further studied.

## Conclusion

Overall, in this study, we found that RAB6B was highly expressed in HCC tissues and was associated with poor prognosis in HCC patients. Furthermore, RAB6B expression promotes the formation of an immunosuppressive microenvironment in HCC through recruiting various immune cells and inducing CD8+T cells exhaustion. Meanwhile, genomic alterations, functional enrichment analysis, and potential targeted drugs of RAB6B were analyzed. *In vitro* experiments have shown that knockdown of RAB6B inhibited cell proliferation, promoted cell apoptosis, and enhanced the sensitivity to cisplatin.

## Data Availability

The datasets presented in this study can be found in online repositories. The names of the repository/repositories and accession number(s) can be found in the article/Supplementary Material.

## References

[B1] Agarwal N. Dancik G. M. Goodspeed A. Costello J. C. Owens C. Duex J. E. (2016). GON4L drives cancer growth through a YY1-androgen receptor-CD24 Axis. Cancer Res. 76, 5175–5185. 10.1158/0008-5472.Can-16-1099 27312530 PMC5010501

[B2] Anand S. Khan M. A. Khushman M. Dasgupta S. Singh S. Singh A. P. (2020). Comprehensive analysis of expression, clinicopathological association and potential prognostic significance of RABs in pancreatic cancer. Int. J. Mol. Sci. 21, 5580. 10.3390/ijms21155580 PMC7432855 32759795

[B3] Asplund A. Edqvist P. H. D. Schwenk J. M. Ponten F. (2012). Antibodies for profiling the human proteome-The Human Protein Atlas as a resource for cancer research. Proteomics 12, 2067–2077. 10.1002/pmic.201100504 22623277

[B4] Beltra J. C. Manne S. Abdel-Hakeem M. S. Kurachi M. Giles J. R. Chen Z. (2020). Developmental relationships of four exhausted CD8(+) T cell subsets reveals underlying transcriptional and epigenetic landscape control mechanisms. Immunity 52, 825–841. 10.1016/j.immuni.2020.04.014 32396847 PMC8360766

[B5] Chandrashekar D. S. Bashel B. Balasubramanya S. a. H. Creighton C. J. Ponce-Rodriguez I. Chakravarthi B. (2017). Ualcan: A portal for facilitating tumor subgroup gene expression and survival analyses. Neoplasia 19, 649–658. 10.1016/j.neo.2017.05.002 28732212 PMC5516091

[B6] Chang H. Jung W. Kim A. Kim H. K. Kim W. B. Kim J. H. (2017). Expression and prognostic significance of programmed death protein 1 and programmed death ligand-1, and cytotoxic T lymphocyte-associated molecule-4 in hepatocellular carcinoma. APMIS 125, 690–698. 10.1111/apm.12703 28493410

[B7] Chaudhuri O. Koshy S. T. Branco Da Cunha C. Shin J. W. Verbeke C. S. Allison K. H. (2014). Extracellular matrix stiffness and composition jointly regulate the induction of malignant phenotypes in mammary epithelium. Nat. Mat. 13, 970–978. 10.1038/nmat4009 24930031

[B8] Chen S. Z. Cao Q. Q. Wen W. Wang H. Y. (2019). Targeted therapy for hepatocellular carcinoma: Challenges and opportunities. Cancer Lett. 460, 1–9. 10.1016/j.canlet.2019.114428 31207320

[B9] Chen X. Song E. (2019). Turning foes to friends: Targeting cancer-associated fibroblasts. Nat. Rev. Drug Discov. 18, 99–115. 10.1038/s41573-018-0004-1 30470818

[B10] Cheng S. Jiang X. Ding C. Du C. Owusu-Ansah K. G. Weng X. (2016). Expression and critical role of interleukin enhancer binding factor 2 in hepatocellular carcinoma. Int. J. Mol. Sci. 17, 1373. 10.3390/ijms17081373 PMC5000768 27556459

[B11] Craig. A. J. Von Felden J. Garcia-Lezana T. Sarcognato S. Villanueva A. (2020). Tumour evolution in hepatocellular carcinoma. Nat. Rev. Gastroenterol. Hepatol. 17, 139–152. 10.1038/s41575-019-0229-4 ( 31792430

[B12] Ejrnaes M. Filippi C. M. Martinic M. M. Ling E. M. Togher L. M. Crotty S. (2006). Resolution of a chronic viral infection after interleukin-10 receptor blockade. J. Exp. Med. 203, 2461–2472. 10.1084/jem.20061462 17030951 PMC2118120

[B13] El Tekle G. Bernasocchi T. Unni A. M. Bertoni F. Rossi D. Rubin M. A. (2021). Co-Occurrence and mutual exclusivity: What cross-cancer mutation patterns can tell us. Trends Cancer 7, 823–836. 10.1016/j.trecan.2021.04.009 34031014

[B14] Elosegui-Artola A. Oria R. Chen Y. Kosmalska A. Perez-Gonzalez C. Castro N. (2016). Mechanical regulation of a molecular clutch defines force transmission and transduction in response to matrix rigidity. Nat. Cell Biol. 18, 540–548. 10.1038/ncb3336 27065098

[B15] Ferlay J. Soerjomataram I. Dikshit R. Eser S. Mathers C. Rebelo M. (2015). Cancer incidence and mortality worldwide: Sources, methods and major patterns in GLOBOCAN 2012. Int. J. Cancer 136, E359–E386. 10.1002/ijc.29210 25220842

[B16] Forbes S. A. Beare D. Gunasekaran P. Leung K. Bindal N. Boutselakis H. (2015). Cosmic: Exploring the world's knowledge of somatic mutations in human cancer. Nucleic Acids Res. 43, D805–D811. 10.1093/nar/gku1075 25355519 PMC4383913

[B17] Fu Y. Liu S. Zeng S. Shen H. (2019). From bench to bed: The tumor immune microenvironment and current immunotherapeutic strategies for hepatocellular carcinoma. J. Exp. Clin. Cancer Res. 38, 396. 10.1186/s13046-019-1396-4 31500650 PMC6734524

[B18] Gao J. Aksoy B. A. Dogrusoz U. Dresdner G. Gross B. Sumer S. O. (2013). Integrative analysis of complex cancer genomics and clinical profiles using the cBioPortal. Sci. Signal. 6, pl1. 10.1126/scisignal.2004088 23550210 PMC4160307

[B19] Gopal Krishnan P. D. Golden E. Woodward E. A. Pavlos N. J. Blancafort P. (2020). Rab GTPases: Emerging oncogenes and tumor suppressive regulators for the editing of survival pathways in cancer. Cancers (Basel) 12, 259. 10.3390/cancers12020259 PMC7072214 31973201

[B20] Hoadley K. A. Yau C. Hinoue T. Wolf D. M. Lazar A. J. Drill E. (2018). Cell-of-Origin patterns dominate the molecular classification of 10, 000 tumors from 33 types of cancer. Cell 173, 291–304. 10.1016/j.cell.2018.03.022 29625048 PMC5957518

[B21] Inagaki Y. Yasui K. Endo M. Nakajima T. Zen K. Tsuji K. (2008). CREB3L4, INTS3, and SNAPAP are targets for the 1q21 amplicon frequently detected in hepatocellular carcinoma. Cancer Genet. cytogenet. 180, 30–36. 10.1016/j.cancergencyto.2007.09.013 18068530

[B22] Jia W. Liang S. Cheng B. Ling C. (2021). The role of cancer-associated fibroblasts in hepatocellular carcinoma and the value of traditional Chinese medicine treatment. Front. Oncol. 11, 763519. 10.3389/fonc.2021.763519 34868982 PMC8636329

[B23] Jiang X. Yang L. Gao Q. Liu Y. Feng X. Ye S. (2022). The role of RAB GTPases and its potential in predicting immunotherapy response and prognosis in colorectal cancer. Front. Genet. 13, 828373. 10.3389/fgene.2022.828373 35154286 PMC8833848

[B24] Lanczky A. Gyorffy B. (2021). Web-based survival analysis tool tailored for medical research (KMplot): Development and implementation. J. Med. Internet Res. 23, e27633. 10.2196/27633 34309564 PMC8367126

[B25] Li G. Marlin M. C. (2015). Rab family of GTPases. Methods Mol. Biol. 1298, 1–15. 10.1007/978-1-4939-2569-8_1 25800828 PMC5903570

[B26] Li L. Ye T. Zhang Q. Li X. Ma L. Yan J. (2021). The expression and clinical significance of TPM4 in hepatocellular carcinoma. Int. J. Med. Sci. 18, 169–175. 10.7150/ijms.49906 33390785 PMC7738955

[B27] Li T. Fan J. Wang B. Traugh N. Chen Q. Liu J. S. (2017). Timer: A web server for comprehensive analysis of tumor-infiltrating immune cells. Cancer Res. 77, e108–e110. 10.1158/0008-5472.CAN-17-0307 29092952 PMC6042652

[B28] Lu C. Rong D. Zhang B. Zheng W. Wang X. Chen Z. (2019). Current perspectives on the immunosuppressive tumor microenvironment in hepatocellular carcinoma: Challenges and opportunities. Mol. Cancer 18, 130. 10.1186/s12943-019-1047-6 31464625 PMC6714090

[B29] Nishida N. Kudo M. (2018). Immune checkpoint blockade for the treatment of human hepatocellular carcinoma. Hepatol. Res. 48, 622–634. 10.1111/hepr.13191 29734514

[B30] Opdam F. J. M. Echard A. Croes H. J. E. Van Den Hurk J. a. J. M. Van De Vorstenbosch R. A. Ginsel L. A. (2000). The small GTPase Rab6B, a novel Rab6 subfamily member, is cell-type specifically expressed and localised to the Golgi apparatus. J. Cell Sci. 113, 2725–2735. 10.1242/jcs.113.15.2725 10893188

[B31] Quail D. F. Joyce J. A. (2013). Microenvironmental regulation of tumor progression and metastasis. Nat. Med. 19, 1423–1437. 10.1038/nm.3394 24202395 PMC3954707

[B32] Ru B. Wong C. N. Tong Y. Zhong J. Y. Zhong S. S. W. Wu W. C. (2019). Tisidb: An integrated repository portal for tumor-immune system interactions. Bioinformatics 35, 4200–4202. 10.1093/bioinformatics/btz210 30903160

[B33] Stenmark H. (2009). Rab GTPases as coordinators of vesicle traffic. Nat. Rev. Mol. Cell Biol. 10, 513–525. 10.1038/nrm2728 19603039

[B34] Tang Z. Kang B. Li C. Chen T. Zhang Z. (2019). GEPIA2: An enhanced web server for large-scale expression profiling and interactive analysis. Nucleic Acids Res. 47, W556–W560. 10.1093/nar/gkz430 31114875 PMC6602440

[B35] Tinoco R. Alcalde V. Yang Y. Sauer K. Zuniga E. I. (2009). Cell-intrinsic transforming growth factor-beta signaling mediates virus-specific CD8+ T cell deletion and viral persistence *in vivo* . Immunity 31, 145–157. 10.1016/j.immuni.2009.06.015 19604493 PMC3039716

[B36] Vasaikar S. V. Straub P. Wang J. Zhang B. (2018). LinkedOmics: Analyzing multi-omics data within and across 32 cancer types. Nucleic Acids Res. 46, D956–D963. 10.1093/nar/gkx1090 29136207 PMC5753188

[B37] Wanschers B. F. Van De Vorstenbosch R. Schlager M. A. Splinter D. Akhmanova A. Hoogenraad C. C. (2007). A role for the Rab6B Bicaudal-D1 interaction in retrograde transport in neuronal cells. Exp. Cell Res. 313, 3408–3420. 10.1016/j.yexcr.2007.05.032 17707369

[B38] Wherry E. J. (2011). T cell exhaustion. Nat. Immunol. 12, 492–499. 10.1038/ni.2035 21739672

[B39] Wong C. C. L. Tse A. P. W. Huang Y. P. Zhu Y. T. Chiu D. K. C. Lai R. K. H. (2014). Lysyl oxidase-like 2 is critical to tumor microenvironment and metastatic niche formation in hepatocellular carcinoma. Hepatology 60, 1645–1658. 10.1002/hep.27320 25048396

[B40] Yang C. C. Meng G. X. Dong Z. R. Li T. (2021). Role of Rab GTPases in hepatocellular carcinoma. J. Hepatocell. Carcinoma 8, 1389–1397. 10.2147/JHC.S336251 34824998 PMC8610749

[B41] Yang F. Wei Y. Han D. Li Y. Shi S. Jiao D. (2020). Interaction with CD68 and regulation of GAS6 expression by Endosialin in fibroblasts drives recruitment and polarization of macrophages in hepatocellular carcinoma. Cancer Res. 80, 3892–3905. 10.1158/0008-5472.CAN-19-2691 32591411

[B42] Yin Z. Dong C. Jiang K. Xu Z. Li R. Guo K. (2019). Heterogeneity of cancer-associated fibroblasts and roles in the progression, prognosis, and therapy of hepatocellular carcinoma. J. Hematol. Oncol. 12, 101. 10.1186/s13045-019-0782-x 31547836 PMC6757399

[B43] Zhang Q. He Y. Luo N. Patel S. J. Han Y. Gao R. (2019). Landscape and dynamics of single immune cells in hepatocellular carcinoma. Cell 179, 829–845. 10.1016/j.cell.2019.10.003 31675496

[B44] Zhao L. Xue M. Zhang L. Guo B. Qin Y. Jiang Q. (2020). MicroRNA-4268 inhibits cell proliferation via AKT/JNK signalling pathways by targeting Rab6B in human gastric cancer. Cancer Gene Ther. 27, 461–472. 10.1038/s41417-019-0118-6 31303644

