# Supplementary figures and images for: RAB6B is a potential prognostic marker and correlated with the remolding of tumor immune microenvironment in hepatocellular carcinoma
Source: Front Pharmacol. 2022 Sep 2;13:989655. doi: 10.3389/fphar.2022.989655 (PMC9478551; doi:10.3389/fphar.2022.989655)

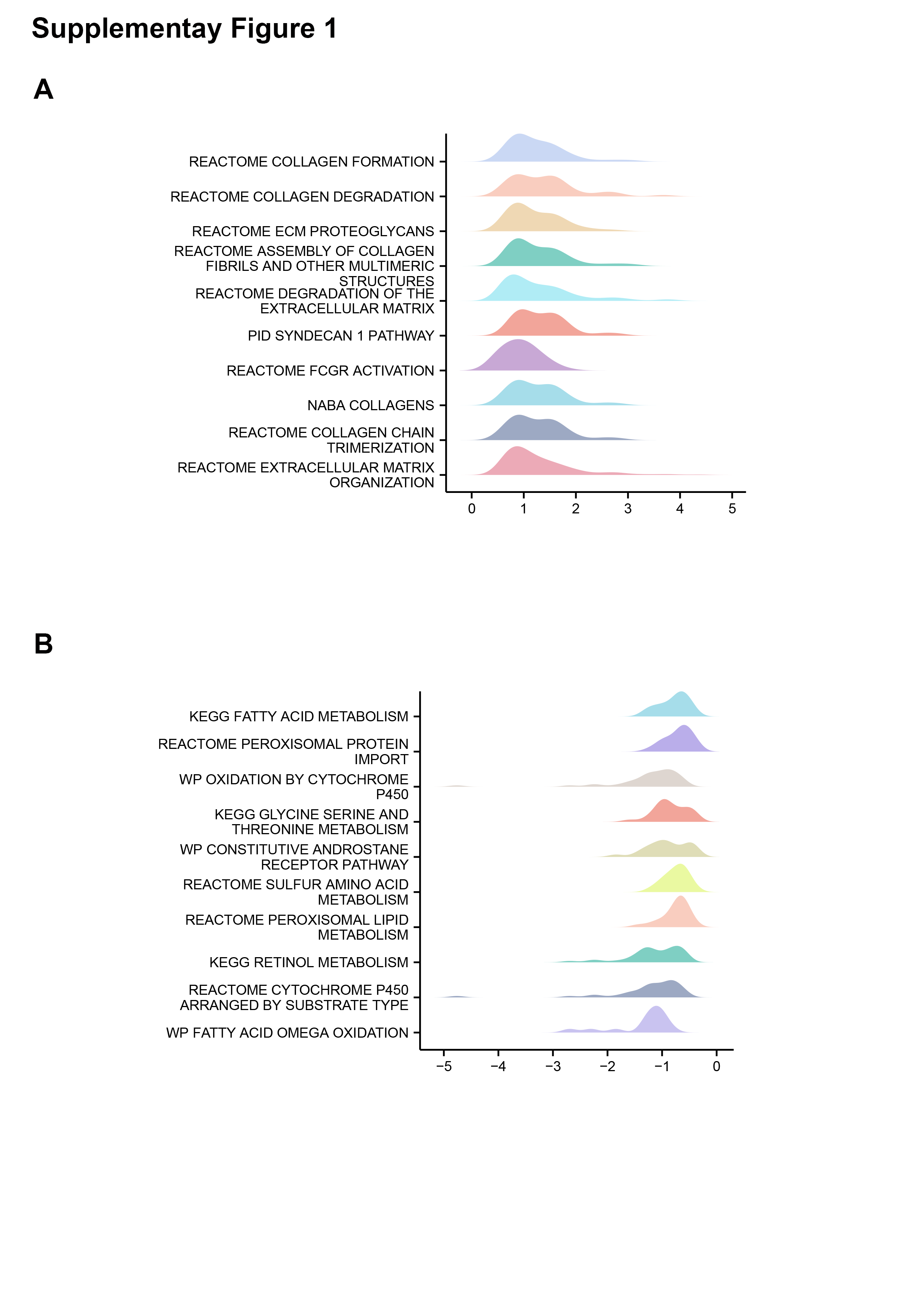

Supplement: Supplementary file 1 [file Image1.TIF]
